# Prospective evaluation of genome sequencing to compare conventional cytogenetics in acute myeloid leukemia

**DOI:** 10.1038/s41408-023-00908-5

**Published:** 2023-09-06

**Authors:** Beth A. Pitel, Cinthya Zepeda-Mendoza, Zohar Sachs, Hongwei Tang, Suganti Shivaram, Neeraj Sharma, James B. Smadbeck, Stephanie A. Smoley, Kathryn E. Pearce, Ivy M. Luoma, Joselle Cook, Mark R. Litzow, Nicole L. Hoppman, David Viswanatha, Xinjie Xu, Rhett P. Ketterling, Patricia T. Greipp, Jess F. Peterson, Linda B. Baughn

**Affiliations:** 1 Department of Laboratory Medicine and Pathology, Division of Laboratory Genetics and Genomics, Mayo Clinic, Rochester, MN USA; 2 Department of Laboratory Medicine and Pathology, Division of Hematopathology, Mayo Clinic, Rochester, MN USA; 3 Division of Hematology, Oncology, and Transplantation, Department of Medicine and Masonic Cancer Center, University of Minnesota, Minneapolis, MN USA; 4 Center for Individualized Medicine-Biomarker Discovery, Mayo Clinic, Rochester, MN USA; 5 Department of Medicine, Division of Hematology, Mayo Clinic, Rochester, MN USA

**Keywords:** Cancer genetics, Cancer genomics

Dear Editors,

Acute myeloid leukemia (AML) can be classified into multiple genetic subtypes based on recurrent pathogenic structural variants (SVs), copy number abnormalities (CNAs), and single nucleotide variants (SNVs) that inform prognostication and clinical management [1, 2]. Karyotype analysis is considered mandatory in the evaluation of AML. If karyotype analysis fails, fluorescence in situ hybridization (FISH) can be used as an alternative technique per ELN 2022 [2]. Recent reports have proposed whole genome sequencing (WGS) as an alternative methodology to karyotyping and FISH [3, 4]. MPseq is one example of a WGS technique optimized for the detection of genome-wide SVs and CNAs [5]. However, previous studies have not directly assessed the prognostic utility of WGS approaches in identifying genetic abnormalities above gold-standard cytogenetic approaches within an unbiased, prospective setting.

Here, we performed a prospective evaluation of MPseq in comparison to karyotyping and FISH combined with panel sequencing in the genetic characterization of 105 cases of AML from the Mayo Clinic from August 2017 to December 2018 (Fig. 1A, Supplementary Figs, 1, 2, Supplementary materials and methods). The median age of the cohort was 65 years (range 1–90) with 10 (9.5%) patients under the age of 30 years. Just over half of the cases represented de novo AML (*n* = 55, 52%), 23 (22%) had AML with myelodysplasia-related changes (AML-MRC), 21 (20%) had relapsed AML and 6 (6%) had therapy related AML (Supplementary Tables 1, 2). The most prevalent cytogenetic result based on all three methodologies was a normal karyotype (37 cases, 35%), followed by deletions of chromosomes 5q and/or 7q (25 cases, 24%). Seven had a simple, non-complex karyotype with non-subtype defining abnormalities, 6 had trisomy 8, 5 had a complex karyotype without 5q or 7q deletions (atypical complex), 6 had a *NUP98* (11p15.4) rearrangement and 7 had a *KMT2A* (11q23.3) rearrangement with gene partner *MLLT10* (10p12.31) in 3 cases, *ELL* (19p13.11) in one case, *MLLT6* (17q12) in one case and *MLLT3* (9p21.3) in 2 cases. Four had t(15;17)(q24;q21), 3 had inv(3)(q21.3q26.2) or t(3;3)(q21.3;q26.2) including a single case with inv(3) with *BCR::ABL1*, 3 had inv(16)(p13.1q22) or t(16;16)(p13.1;q22), and a single case each with either t(6;9)(p23;q34.1) or *KAT6A* rearrangement involving 8p11.2 (Fig. 1B).

**Fig. 1:**
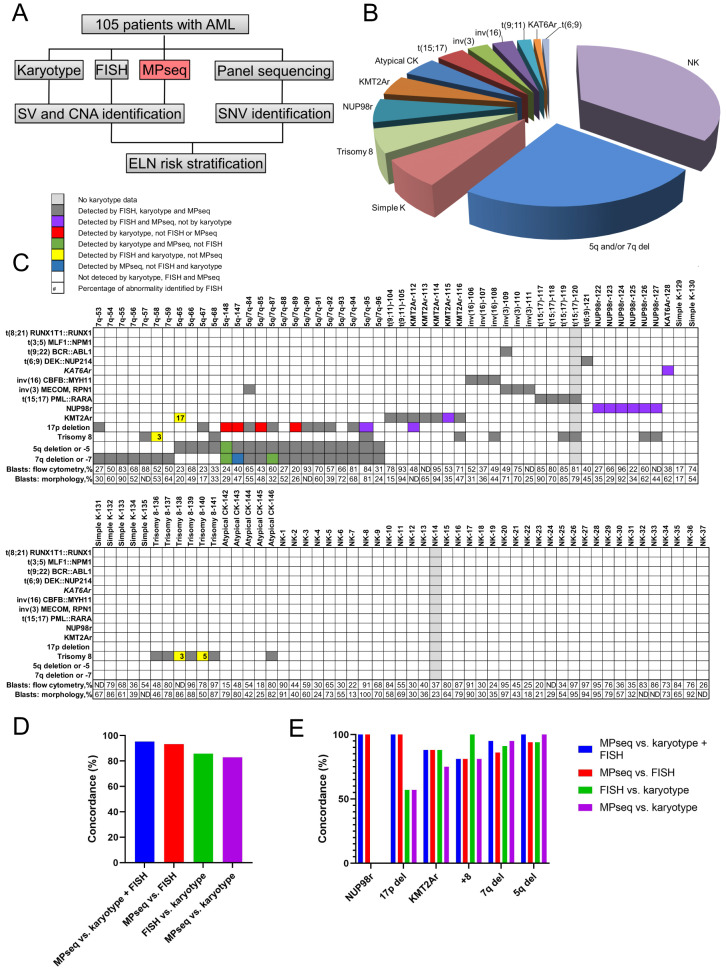
Genetic characterization and abnormal distribution of AML cohort. **A** 105 cases from patients with a diagnosis of AML. Karyotype, FISH, and MPseq was performed to identify the structural variation (SV) including copy number abnormality (CNA), results were tabulated, and cases were divided into the following subtypes based on karyotype, FISH, or MPseq results. NGS-based panel sequencing was also performed to identify pathogenic or likely pathogenic single nucleotide variants (SNVs). **B** Pie chart displaying the relative distribution for each cytogenetic subtype in 105 AML cases. Normal Karyotype (NK, 35%), 5q deletion (5q del) and/or 7q deletion (7q del) (24%), simple karyotype (7%), Trisomy 8 (6%), *NUP98* rearrangement (NUP98r, 6%), *KMT2A* rearrangement excluding t(9;11)(p22;q23) (5%), atypical complex karyotype (CK) (5%), t(15;17)(q24;q21) (4%), inv(3)(q21.3q26.2) (3%), inv(16)(p13.1q22) (3%), t(9;11)(p22;q23) (2%), t(6;9)(p23;q34.1) (1%) and *KAT6A* rearrangement (1%). The t(9;11) rearrangements are separated from other *KMT2A* rearrangements due to their differential influence on outcome. **C** Distribution of discrepant cases are indicated in the boxes. No karyotype data are indicated in light grey. Abnormalities detected by FISH, karyotype and MPseq are indicated in dark grey, abnormalities detected by FISH and MPseq but not karyotype are indicated in purple, abnormalities detected by karyotype but not FISH or MPseq are indicated in red, abnormalities detected by karyotype and MPseq but not FISH are indicated in green, abnormalities detected by FISH and karyotype, but not MPseq are indicated in yellow and abnormalities detected by MPseq, but not FISH and karyotype are indicated in blue. Abnormalities not detected by karyotype, FISH and MPseq are indicated in white. Blast by flow cytometry or morphology are indicated. **D** Percentage of concordance between MPseq vs. karyotype + FISH, MPseq vs. FISH, FISH vs. karyotype and MPseq vs. karyotype for AML-related genomic events described in **C**. **E** Percentage of concordance between each MPseq vs. karyotype + FISH, MPseq vs. FISH, FISH vs. karyotype, and MPseq vs. karyotype for individual genomic events.

When evaluating cytogenetic results obtained by MPseq compared to karyotype plus FISH, 100 of 105 cases were concordant (95.2%) (Table 1, Fig. 1C, D). Of the 5 discordant cases, MPseq missed 4 abnormalities that were identified by karyotype and FISH. All 4 were found in a low level by FISH including a *KMT2A* rearrangement detected in 17% of cells in a case with t(11;17)(q23;q11.2). In 3 other cases, MPseq missed low-level trisomy 8 found in 3–5% of cells by FISH and detected by karyotype (Table 1, Fig. 1C, D). Since MPseq is validated to identify SVs >10% and CNAs >25% of the tumor clone [5], the missed *KMT2A*r represents a false negative result. The final case of discordance included an atypical 7q deletion identified by MPseq that was missed by karyotype and FISH because it did not map within the FISH probe utilized (Table 1).

**Table 1:** Cases with discrepancies between karyotype, FISH and MPseq.

Case ID	Abnormality	Karyotype	FISH	MPseq	Concordance between MPseq vs. FISH+Karyotype
5q/7q-85	5q del	Y	Y	Y	Y
	7q del	Y	Y	Y	Y
	*TP53* del	Predicted del	N	N	Y
5q/7q-87	5q del	Y	Y	Y	Y
	7q del	Y	Missed	Y	Y
5q/7q-89	5q del	Y	Y	Y	Y
	7q del	Y	Y	Y	Y
	*TP53* del	Predicted del	N	N	Y
5q/7q-95	5q del	Y	Y	Y	Y
	7q del	Y	Y	Y	Y
	*TP53* del	Missed	Y	Y	Y
KMT2Ar-112	*KMT2Ar*	Y	Y	Y	Y
	*TP53*del	Missed	Y	Y	Y
KMT2Ar-115	*KMT2Ar*	Missed	Y	Y	Y
KAT6Ar-128	*KAT6Ar*	Missed	Y	Y	Y
NUP98/KDM5A-122	*NUP98r*	Missed	Y	Y	Y
NUP98/KDM5A-123	*NUP98r*	Missed	Y	Y	Y
NUP98/KDM5A-124	*NUP98r*	Missed	Y	Y	Y
NUP98/KDM5A-125	*NUP98r*	Missed	Y	Y	Y
NUP98/NSD1-126	*NUP98r*	Missed	Y	Y	Y
NUP98/NSD1-127	*NUP98r*	Missed	Y	Y	Y
5q-65	5qdel	Y	Y	Y	Y
	*KMT2Ar*	Y	Y	Missed	N
Trisomy 8-138	Trisomy 8	Y	Y	Missed	N
Trisomy 8-140	Trisomy 8	Y	Y	Missed	N
7q-58	7q del	Y	Y	Y	Y
	Trisomy 8	Y	Y	Missed	N
5q-147	5q del	Y	Y	Y	Y
	7q del	Missed	Missed	Y	N
	*TP53* del	Predicted del	N	N	Y
5q-148	5q del	Y	Missed	Y	Y
	7q del	Y	Missed	Y	Y
	*TP53* del	Predicted del	N	N	Y

All cases with evidence of discordance between FISH, karyotype and MPseq. A direct comparison of concordance between data obtained from FISH along with karyotype vs. MPseq. Data from karyotype and FISH are combined to indicate the complementary testing approaches. Note: Y for yes; N for No.

When evaluating cytogenetic results obtained by each methodology individually, the concordance between MPseq and FISH was 93.3%, between FISH and karyotype was 85.7% and between MPseq and karyotype was 82.9% (Fig. 1D). FISH missed 4 abnormalities identified by MPseq involving deletions of 5q in 1 case and 7q in 3 cases because the deletions did not map within the FISH probe utilized (Table 1, Supplementary Fig. 3). Karyotype analysis failed to detect 11 abnormalities that were identified by FISH or MPseq (Table 1). These included 6 cases with a *NUP98* rearrangement, 2 cases with a *TP53* deletion, and one case each with a *KMT2A::MLLT10* fusion and an inv(8) resulting in a *KAT6A* rearrangement, and a 7q deletion. Additionally, karyotype analysis detected 4 cases with a 17p deletion, which presumptively included loss of the *TP53* locus, that were not identified by FISH or MPseq. There was 100% concordance for some AML-associated rearrangements with consistent and cytogenetically detectable breakpoints including t(9;22)(q34.1;q11.2), t(6;9)(p23.3;q34.1), inv(16)(p13.1q22), inv(3)(q21.3q26.2), and t(15;17)(q24;q21) (Fig. 1C) demonstrating that karyotype, FISH, and MPseq are similarly reliable methodologies to identify these recurrent rearrangements. However, as expected, all *NUP98* rearrangements observed in this study were cryptic by karyotype and detected by both FISH and MPseq (Fig. 1E). Reduced concordance between karyotype and MPseq was also observed for 17p/*TP53* deletions (57%), *KMT2A* rearrangements (75%) and trisomy 8 (81%) (Fig. 1E). FISH and MPseq have a higher sensitivity in comparison to karyotype analysis in detecting *NUP98* and *KMT2A* rearrangements and *TP53* deletions. For low-level abnormalities (<25%), FISH has increased sensitivity compared to MPseq if the abnormality is targetable by the available FISH probe.

Of the 5 cases with discordant results between MPseq compared to karyotype plus FISH (Table 1), the ELN risk stratification remained unchanged (Supplementary Table 3). No additional cryptic, prognostically defining genetic abnormalities per ELN 2022 were identified by MPseq in the remaining samples, including those with a normal or simple karyotype (Fig. 1C). Thus, MPseq did not alter the ELN risk stratification above information provided by karyotype combined with FISH demonstrating that by using the current risk stratification guidelines, karyotype in combination with FISH analysis remains a robust laboratory approach in the evaluation of AML.

We next evaluated whether MPseq could uncover AML-associated abnormalities not currently incorporated into the ELN guidelines. Using a list of 109 genes implicated in AML or MDS [6], we identified an average of 7.5 individual gene aberrations per case including an average of 3.9 losses, 3.1 gains or amplifications and 0.5 SVs (Supplementary Fig. 4). Overall, MPseq identified additional aberrations in 43 cases (40.1%) involving AML genes undetected by karyotyping (Supplementary Table 4). The most frequently deleted genes included *KMT2C, CUX1,* and *EZH2*, located on 7q and the most frequently gained genes included *MYC* and/or *CCDC26*, *RAD21,* and *TRPS1*, located on chromosome 8. *MECOM* was the most frequently rearranged gene in our AML cohort (Supplementary Figs 4–5). MPseq identified 11 cases (10.5%) with significant regions of gain that are not currently prognostic per the current ELN guidelines including a case (NK-34) of a *KMT2A* partial tandem duplication (Supplementary Fig. 6A), a case (Simple K-129) with 1–28 double minute chromosomes (extrachromosomal circular DNA fragments) with amplification of the *MYC* gene and 3 cases (NK-11, NK-19, 7q-55) with focal gains identified by MPseq of 8q24 involving *MYC* and/or the nearby long noncoding RNA *CCDC26*. The partial gain of *CCDC26* in 7q-55 was also associated with a cryptic ins(14;8)(q32.2;24.21) resulting in an insertion between *BCL11B* into the *CCDC26*. We also observed 2 cases with iAMP21 by MPseq (case 5q-65 and 5q-147) (Supplementary Fig. 6B). Finally, 2 cases (5q/7q-94 and 5q/7q-96) had amplifications of both *MECOM* (with a rearrangement) and *KRAS*. Rare or novel SVs were identified in 20 cases (19.0%) including case *KAT6A*r-128, which was found to have a cryptic inv(8) resulting in a *KAT6A::SORBS3* fusion. A single case (simple K-132) had a *ZMYND11::MBTD1* fusion. SVs disrupting *NF1* were found in 2 cases (Atypical CK-146, 5q/7q-92), *RUNX1* in 2 cases (5q-147, 5q/7q-85), non-*GATA2 MECOM* rearrangements in 6 cases (7q-56, 5q-148, 5q/7q-84, 5q/7q-94, 5q/7q-96, *KMT2A*-112) and an *ASXL1* rearrangement resulting in a partial deletion of the gene in one case (5q/7q-87). Finally, MPseq characterized each of 6 *NUP98* rearrangements revealing *KMD5A* partner gene in 3 cases and *NSD1* partner in 3 cases. Recurrent focal deletions of AML associated genes not appreciated by karyotyping were common (Supplementary Figs 4, 5). Excluding genes located on chromosomes 5 and 7 and *TP53*, the most frequently deleted gene was *NF1* in 9 cases.

In summary, we identified five cases (4.8%) of discordance between MPseq compared to karyotype with FISH when evaluating recurrent AML-associated genetic abnormalities. Greater discordance was identified when comparing karyotype to FISH (14.3%) or karyotype to MPseq (17.1%) individually. These findings contrast previous studies which have reported limited value of FISH in AML when an adequate karyotype is available [7, 8]. Six of our discordant cases included *NUP98* rearrangements, which are often cryptic and not evaluated in previous studies. Thus, for specific aberrations that are difficult to detect karyotypically such as *TP53* deletions and *NUP98* rearrangements, the added value of MPseq is not strong if a comprehensive FISH panel including probes targeting *NUP98* (especially in pediatric AML), *TP53* and *KMT2A* are used. Alterations in *TP53* are associated with significantly inferior outcomes and treatment responses in AML and biallelic alteration in *TP53* AML confers the worse outcomes among CK AMLs [9]. Therefore, accurate characterization of the *TP53* locus is important for prognostication and may soon impact clinical management.

Although we demonstrate that MPseq does not currently add additional prognostic value above FISH when compared to a comprehensive AML FISH panel, risk stratification guidelines will likely evolve to include novel abnormalities of prognostic significance detected by NGS. For example, growing evidence suggests *NUP98* rearrangements are associated with poor outcome [10], but *NUP98* rearrangements have not yet been incorporated into ELN guidelines. Similarly, *KMT2A* partial tandem duplications have been reported in 10% of AML and MDS and are associated with poor outcome [11]. Other rare abnormalities not incorporated into ELN guidelines identified here include *MYC* amplification, associated with a CK and poor outcome [12], *BCL11B::MYC* rearrangements reported as a driver in ambiguous leukemia [13], *ZMYND11::MBTD1* fusion [14] and iAMP21 AML [15].

We demonstrate that karyotype analysis when combined with FISH remains a robust laboratory approach in the evaluation of AML. While the use of MPseq did not add significant value above FISH using current ELN guidelines, NGS technologies such as WGS will continue to identify novel high-risk AML subgroups, which will likely enhance future risk stratification guidelines.

### Supplementary information

Supplementary Methods

Supplementary Figures

Supplementary Tables

Supplementary Table 4

## Data Availability

The datasets generated during and/or analyzed during the current study are available from the corresponding author on reasonable request.
