# Supplementary material for: Prospective evaluation of genome sequencing to compare conventional cytogenetics in acute myeloid leukemia
Source: Blood Cancer J. 2023 Sep 6;13(1):138. doi: 10.1038/s41408-023-00908-5 (PMC10482828; doi:10.1038/s41408-023-00908-5)
Supplement: Supplementary file 1 — Supplementary Methods [file 41408_2023_908_MOESM1_ESM.docx]

**Supplementary Materials and Methods**

**Patient samples**

All samples were obtained and evaluated as part of a research study approved by the Mayo Clinic Institutional Review Board. Samples from 116 consecutive patients of any age diagnosed with AML at the Mayo Clinic from August 2017 to December 2018 were prospectively collected. DNA was extracted from residual bone marrow (BM), blood or fixed cell pellets previously used for cytogenetic studies. Of the 116 patient cases, 11 samples (9.5%) had insufficient DNA for MPseq, resulting in exclusion from the study. SNV information from clinical targeted NGS panels was collected using the Mayo Clinic Genomics Laboratory information system (LIS). Mayo Clinic electronic medical records were queried for confirmation of AML using the WHO 4^th^ Edition classification and determination of de novo or therapy-related AML or AML resulting from evolution from another myeloid neoplasm. A subset of the samples with either a normal karyotype or 5q deletion and/or 7q deletion has been described previously ^1^.

**Karyotype Analysis**

BM cells were cultured, harvested and banded utilizing standard cytogenetic techniques as previously reported ^2^. Briefly, BM was cultured without mitogens in PB-MAX media for 24 or 48 hours and treated with colcemid. Cells were harvested and fixed in an ethanol/glacial acetic acid solution. Slides were prepared and stained for G-banded karyotype analysis using standard (**G**-banding, **T**rypsin treatment and **L**eishman stain) GTL banding cytogenetic procedures. When available, 20 metaphases were analyzed, and results reported per 2016 International System for Human Cytogenomic Nomenclature (ISCN) ^3^. Simple karyotype was defined as < 3 unrelated chromosome abnormalities in the absence of other class-defining recurring genetic abnormalities. Atypical complex karyotype was defined as having > 3 unrelated chromosome abnormalities not involving chromosomes 5 and 7 in the absence of other class-defining recurring genetic abnormalities.

**Fluorescence *in situ* hybridization (FISH)**

BM aspirate specimens were harvested with or without prior culturing, and slides were prepared from the fixed cell suspension. Slides were pretreated using traditional cytogenetic methods and probes were hybridized to specific segments of DNA within the cells on the slide. DAPI was used to stain all nuclei. FISH analysis included evaluation for deletions of 5q, 7q and 17p; gain of chromosome 8; rearrangements of *KMT2A* and *NUP98*; and several translocations and/or inversions: inv(3) or t(3;3)(*GATA2::MECOM*), t(6;9)(*DEK*::*CAN*), t(8;21)(*RUNX1T1*::*RUNX1*), t(15;17)(*PML*::*RARA*), t(8;16)(*KAT6A*::*CREBBP*), inv(16) or t(16;16)(*CBFB::MYH11*) t(9;22)(*ABL1*::*BCR*) and t(3;5)(*MLF1*::*NPM1*) ^2^ (**Supplementary Figure 1**).

**DNA extraction, MPseq, SV, and CNA detection**

DNA extraction and MPseq library preparation methods have been previously described ^1, 2^. Briefly, DNA was isolated using the Qiagen Puregene extraction kit (for blood and BM samples < 2 mL), Autopure (for blood and bone marrow samples > 2mL), and QiaCube (for fixed cell pellet extraction). DNA was processed using the Illumina Nextera Mate Pair library preparation kit (Illumina) and sequenced on the Illumina HiSeq 2500 in rapid run mode as described in Aypar, et al. ^2^. Pooled libraries were hybridized onto a flow cell (2 samples per lane) and sequenced using 101-basepair reads and paired end sequencing. MPseq data were mapped to the reference genome (GRCh38) using BIMA ^4^ and the output was analyzed using SVAtools ^2, 5, 6^. SVs greater than 30 Kb that involve breakpoint junctions and/or CNAs were graphically illustrated using genome, junction and region plots as previously described ^2, 5^. SVs include balanced and unbalanced traslocations, inversions and insertions and CNAs include deletions, gains or amplifications ^7^. Since the sequencing depth of MPseq is shallow, this methodology cannot replace panel sequencing to detect SNVs and small indels. Therefore, SNVs were not assessed by MPseq.

**Panel sequencing for SNV assessment**

Approximately 200 ng of genomic DNA sheared to approximately 150 base-pairs was utilized for library preparation by the SureSelect XT library kit (Agilent, Santa Clara, CA) and sequenced on the HiSeq platform (Illumina, San Diego, CA) using 2x101 base-pair read lengths. NGS data were processed through a bioinformatics pipeline (Mayo NGS Workbench) for alignment, base calling and insertion/deletion (indel) detection. Results were confirmed by reviewing the BAM files in Alamut Visual (Interactive Biosoftware, Rouen, France). Sensitivity was 5−10% variant allele fraction (VAF) with a minimum depth coverage of 250X for the NGS assay. The genes assayed in clinical NGS panels are detailed in **Supplementary Figure 1**.

**References**

1. Pitel BA, Sharma N, Zepeda-Mendoza C, Smadbeck JB, Pearce KE, Cook JM*, et al.* Myeloid malignancies with 5q and 7q deletions are associated with extreme genomic complexity, biallelic TP53 variants, and very poor prognosis. *Blood Cancer Journal* 2021 Feb 8; **11**(2).

2. Aypar U, Smoley SA, Pitel BA, Pearce KE, Zenka RM, Vasmatzis G*, et al.* Mate pair sequencing improves detection of genomic abnormalities in acute myeloid leukemia. *Eur J Haematol* 2019 Jan; **102**(1)**:** 87-96.

3. *An International System for Human Cytogenomic Nomenclature*, 2016.

4. Drucker TM, Johnson SH, Murphy SJ, Cradic KW, Therneau TM, Vasmatzis G. BIMA V3: an aligner customized for mate pair library sequencing. *Bioinformatics* 2014 Jun 1; **30**(11)**:** 1627-1629.

5. Gaitatzes A, Johnson SH, Smadbeck JB, Vasmatzis G. Genome U-Plot: a whole genome visualization. *Bioinformatics* 2018 May 15; **34**(10)**:** 1629-1634.

6. Smadbeck JB, Johnson SH, Smoley SA, Gaitatzes A, Drucker TM, Zenka RM*, et al.* Copy number variant analysis using genome-wide mate-pair sequencing. *Genes Chromosomes Cancer* 2018 Sep; **57**(9)**:** 459-470.

7. Huddleston J, Chaisson MJP, Steinberg KM, Warren W, Hoekzema K, Gordon D*, et al.* Discovery and genotyping of structural variation from long-read haploid genome sequence data. *Genome Res* 2017 May; **27**(5)**:** 677-685.
