# Supplementary material for: Prospective evaluation of genome sequencing to compare conventional cytogenetics in acute myeloid leukemia
Source: Blood Cancer J. 2023 Sep 6;13(1):138. doi: 10.1038/s41408-023-00908-5 (PMC10482828; doi:10.1038/s41408-023-00908-5)
Supplement: Supplementary file 2 — Supplementary Figures [file 41408_2023_908_MOESM2_ESM.pdf]

NGAML - NGS, AML, 8-gene panel

CEBPA
FLT3
IDH1
IDH2
KRAS
NPM1
NRAS
TP53

**genes in 1 NGS test**
**genes in 2 NGS tests**
**genes in 3 NGS tests**

NGSAM - NGS, AML, 19-gene panel

ASXL1	*KIT*	*U2AF1*
BCOR	*NPM1*	*WT1*
CEBPA	*PHF6*	*ZRSR2*
DNMT3A	*RUNX1*	
EZH2	*SF3B1*	
FLT3	*SRSF2*	
IDH1	*TET2*	
IDH2	*TP53*	

NGSHM - NGS, Heme Neoplasms, 35-gene panel

ASXL1	*ETV6*	*KIT*	*PTPN11*	*U2AF1*
BCOR	*EZH2*	*KRAS*	*RUNX1*	*WT1*
BRAF	*FLT3*	*MPL*	*SETBP1*	*ZRSR2*
CALR	*GATA1*	*MYD88*	*SF3B1*	
CBL	*GATA2*	*NOTCH1*	*SRSF2*	
CEBPA	*IDH1*	*NPM1*	*TERT*	
CSF3R	*IDH2*	*NRAS*	*TET2*	
DNMT3A	*JAK2*	*PHF6*	*TP53*	

AML FISH panel

Abnormality Name
Monosomy 5 or deletion 5q31 (D5S630x2,EGR1x1)
Monosomy 7 or deletion 7q31 (D7Z1x2,D7S486x1)
Monsomy 17 or deletion 17p13.1(TP53x1,D17Z1x2)
Trisomy 8 (D8Z2,*MYC*)x3
t(6;9)(p23.3;q34.1) *DEK::NUP214*
t(8;21)(q22;q22.1) *RUNX1T1::RUNX1*
t(15;17)(q24;q21) *PML::RARA*
Reflex: 17q21 rearrangement, *RARA* break-apart
inv(16)(p13.1q22)/t(16;16)(p13.1;q22) *MYH11::CBFB*
t(8;16)(p11.2;p13.3) *KAT6A::CREBBP*
t(9;22)(q34.1;q11.2) *ABL1::BCR*
t(3;5)(q25.3;q35.1) *MLF1::NPM1*
11p15.4 (*NUP98* r)
inv(3)(q21.3q26.2)/t(3;3)(q21.3;q26.2) *GATA2::MECOM*
11q23.3 (*KMT2A* r)
Reflex: t(4;11)(q21;q23), *AFF1::KMT2A*
Reflex: t(6;11)(q27;q23), *MLLT4::KMT2A*
Reflex: t(9;11)(p22;q23), *MLLT3::KMT2A*
Reflex: t(10;11)(p13;q23), *MLLT10::KMT2A*
Reflex: t(11;16)(q23;p13.3), *KMT2A::CREBBP*
Reflex: t(11;19)(q23;p13.1), *KMT2A::ELL*
Reflex: t(11;19)(q23;p13.3), *KMT2A::MLLT1*

Supplementary Figure 1: NGS and FISH panels used in this study

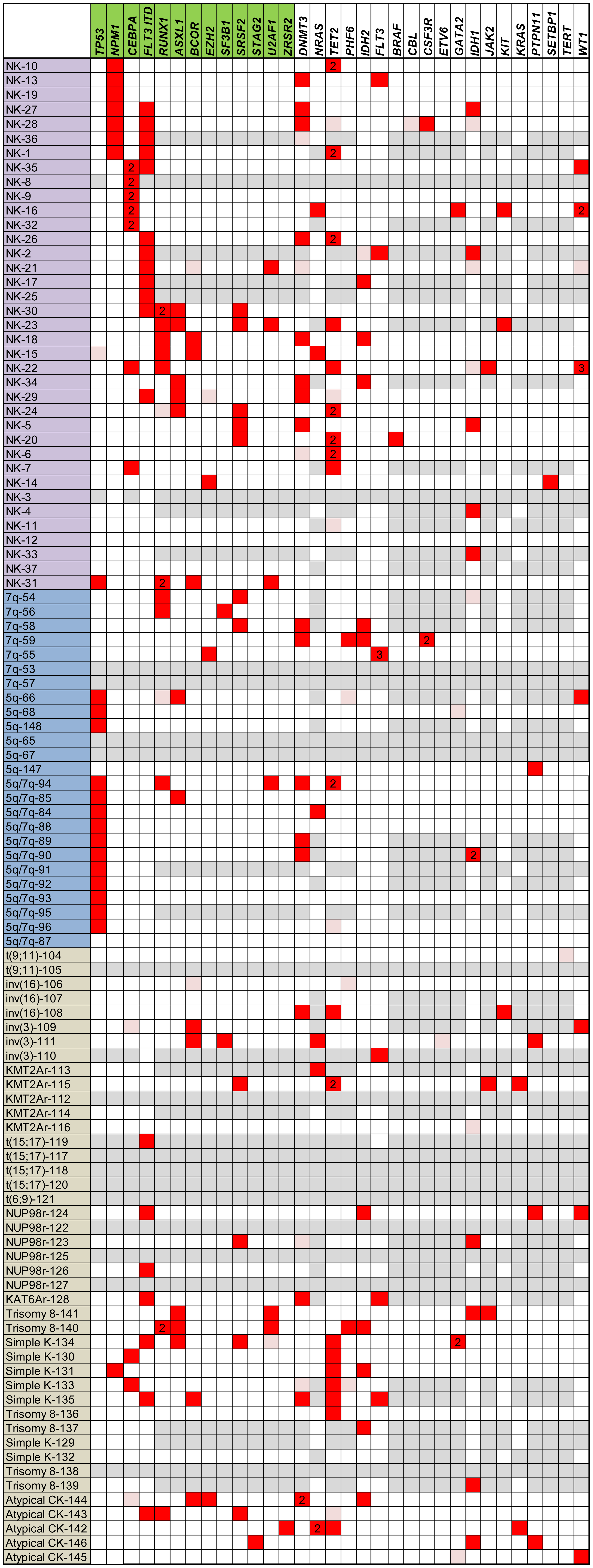

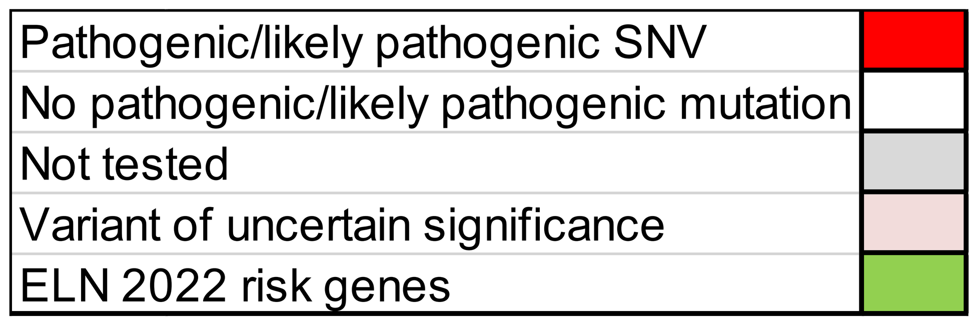

Supplementary Figure 2: Distribution of SNVs in cohort. Genes with pathogenic or likely pathogenic SNVs are annotated in red, no evidence of a pathogenic or likely pathogenic mutations in white, not tested in grey and variant of uncertain significance in pink. Genes included in ELN 2022 guidelines are in green ((*TP53, NPM1, CEPBA, FLT3, RUNX1*, *ASXL1, BCOR, EZH2, SF3B1, SRSF2, STAG2, U2AF1 and ZRSR2)*. When a number is represented within a red box, it indicates the number of pathogenic or likely pathogenic variants identified in a gene.

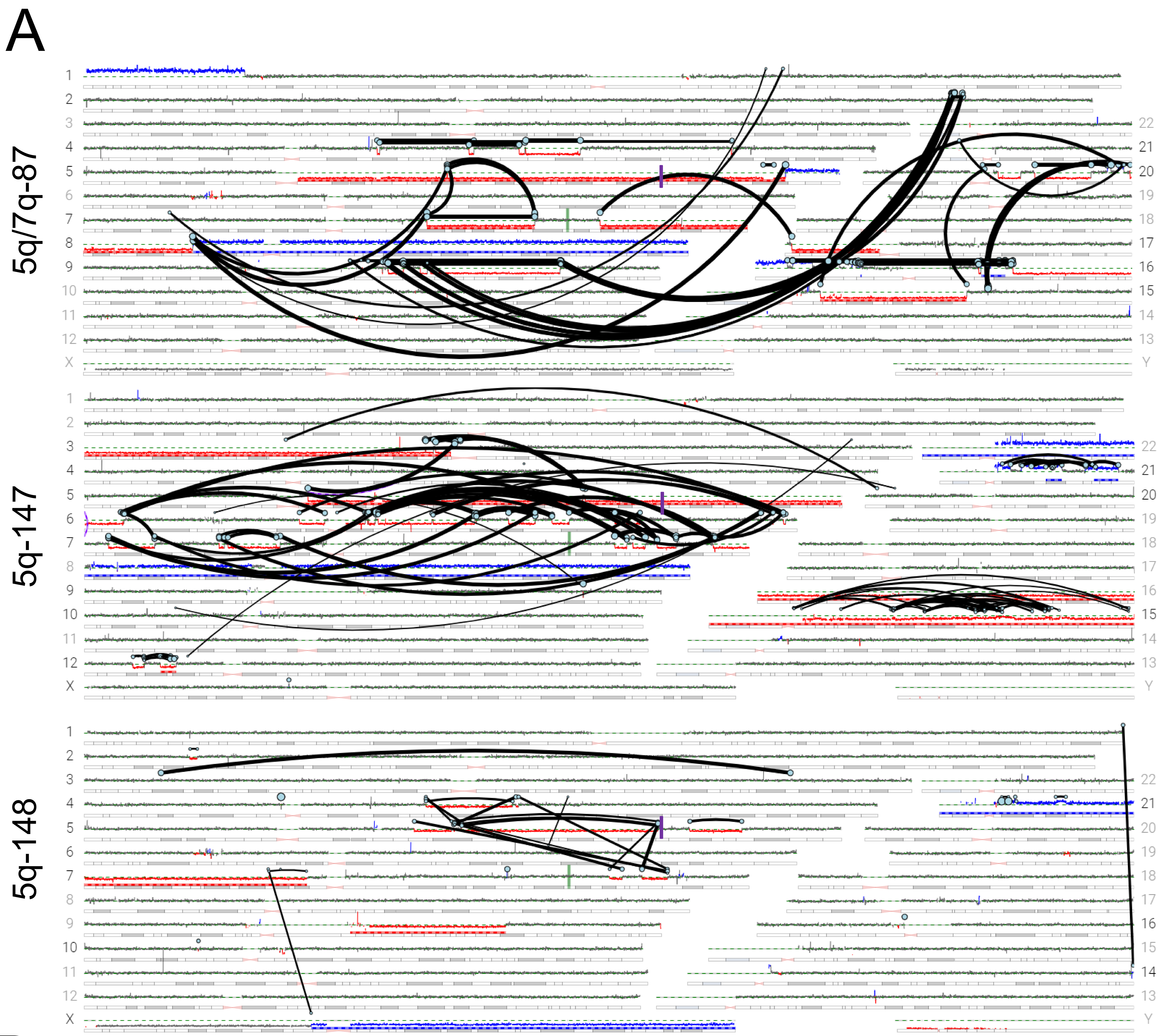

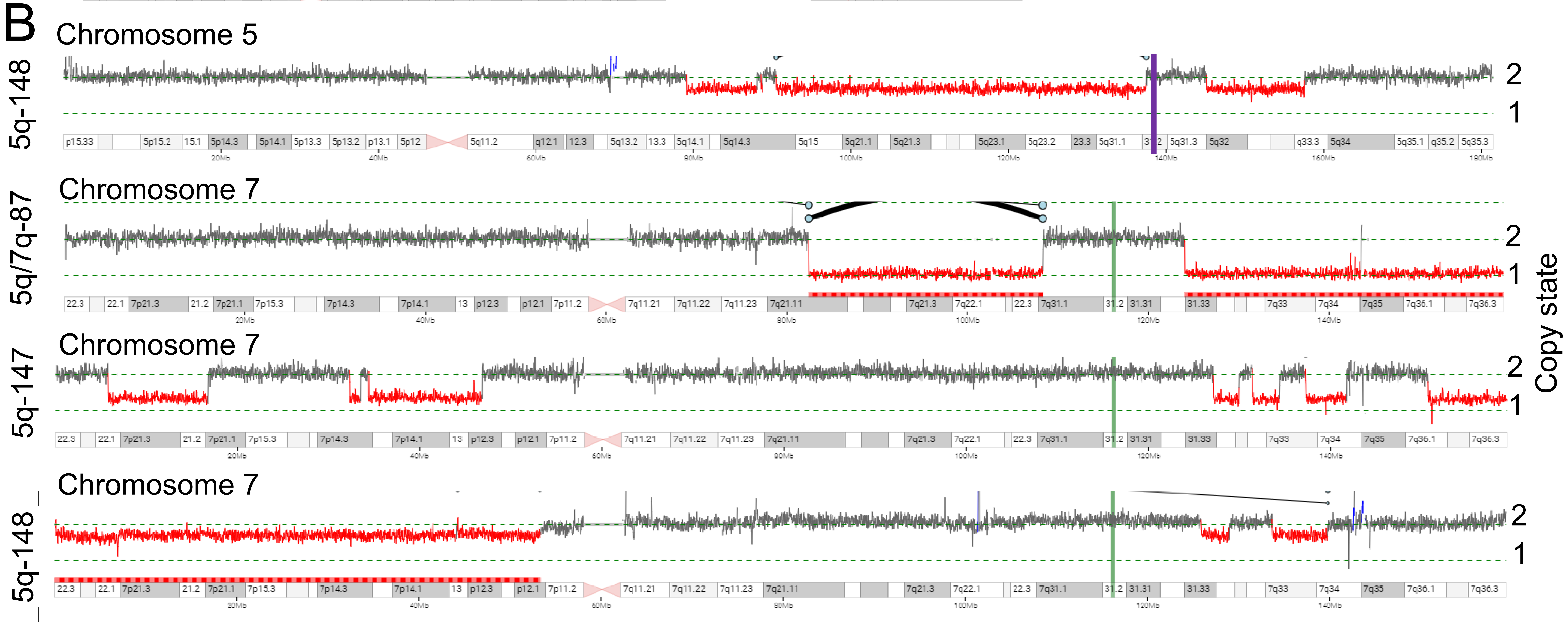

Supplementary Figure 3: Missed 5q and 7q deletions by FISH detected by MPseq. Genome view of the SVs and CNAs in cases with missed FISH abnormalities in **A** and focused views of chromosome 5 and 7 in **B**. Purple line indicates FISH footprint for D5S630/EGR1 chr5:138318520-138527049 and green line indicates FISH footprint for D7S486 chr7:116,035,313-116,343,414 (both GRCh38). Red line indicates copy number losses and blue lines indicate copy number gains, black lines indicate junctions between chromosomes. Copy state (1, 2) is indicated on the right of the image. For 5q-148, FISH missed the 5q del since the region did not include the FISH footprint (purple). For 5q/7q-87, 5q-147, 5q-148, FISH missed the 7q del since the region did not include the FISH footprint (green).

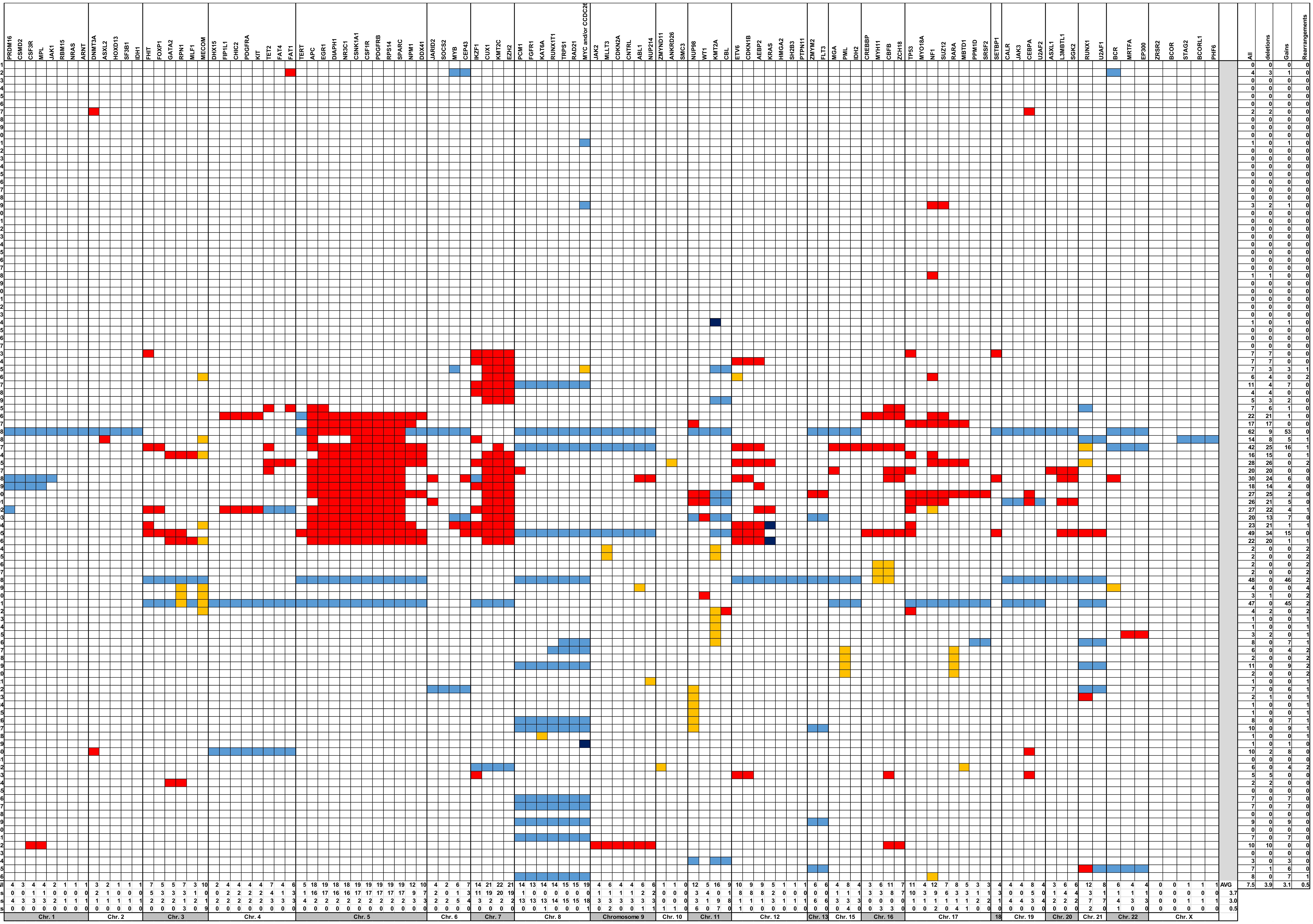

Supplementary Figure 4: Presence or absence or type of abnormality in 109 AML associated genes.
Red: deletions, blue: gains, dark blue: amplifications, yellow: rearrangements.

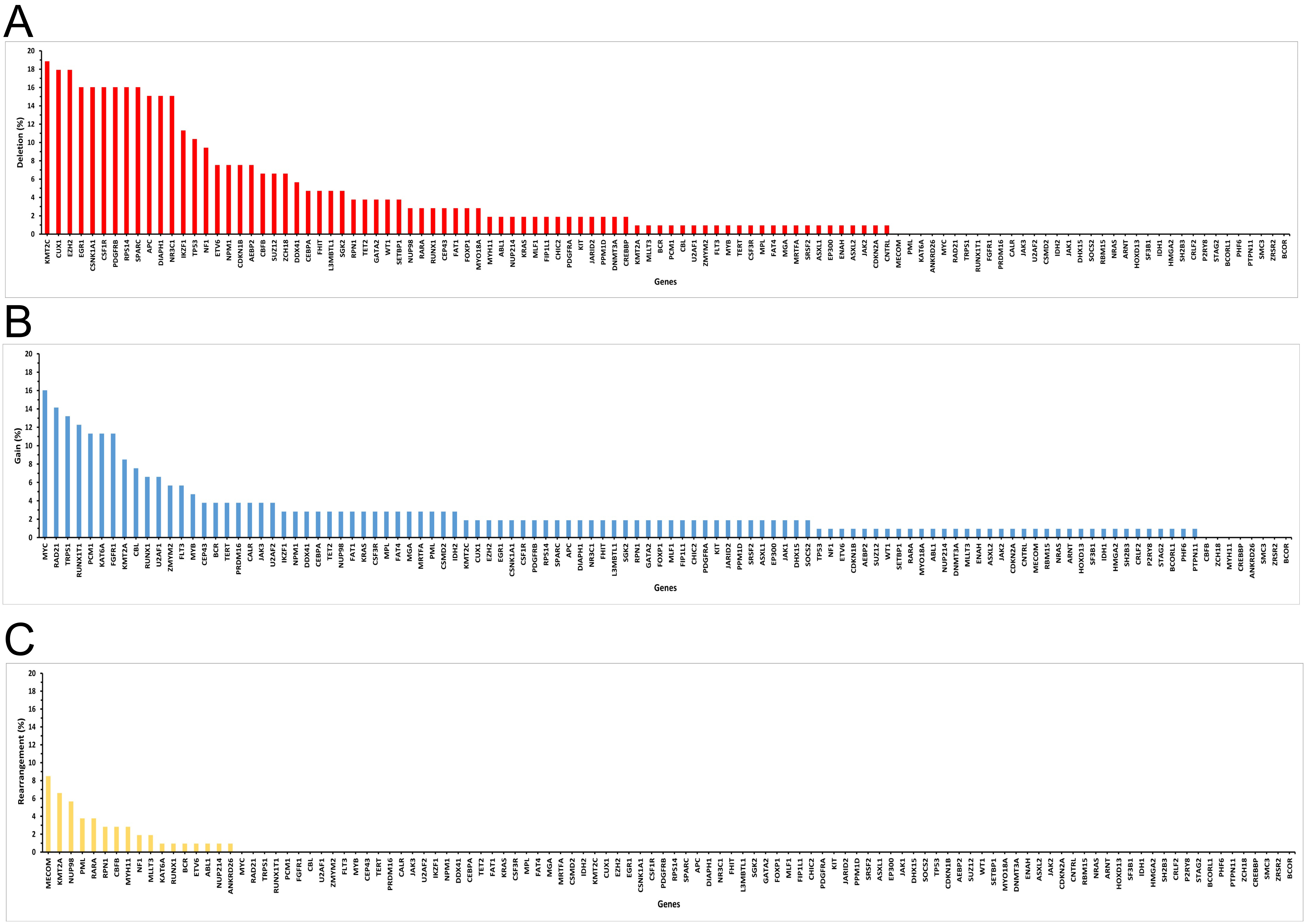

Supplementary Figure 5: Recurrent deletions, gains and rearrangements. Percentage of cases with a deletion in **A**, a gain in **B** and rearrangement in **C**.

A

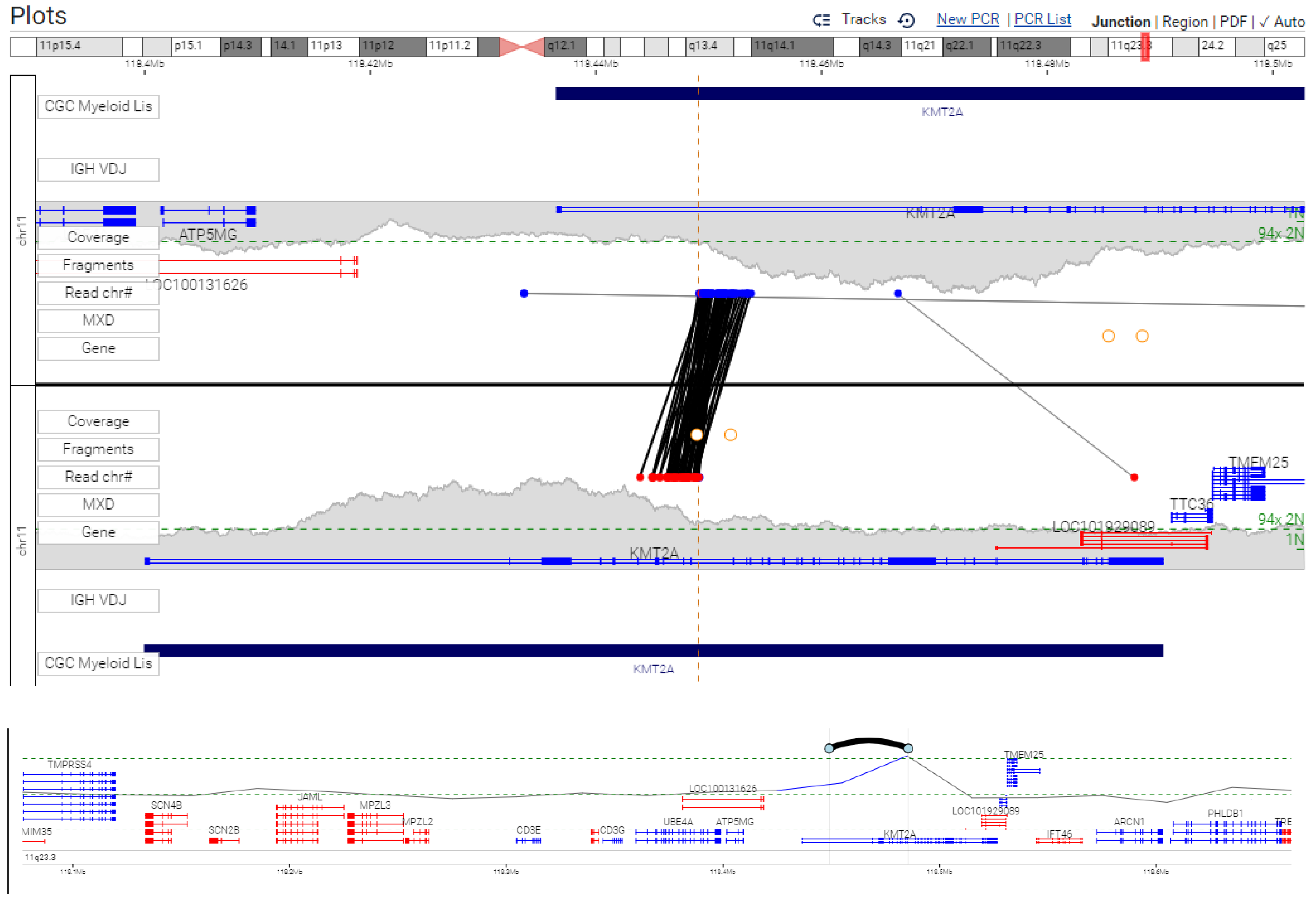

B

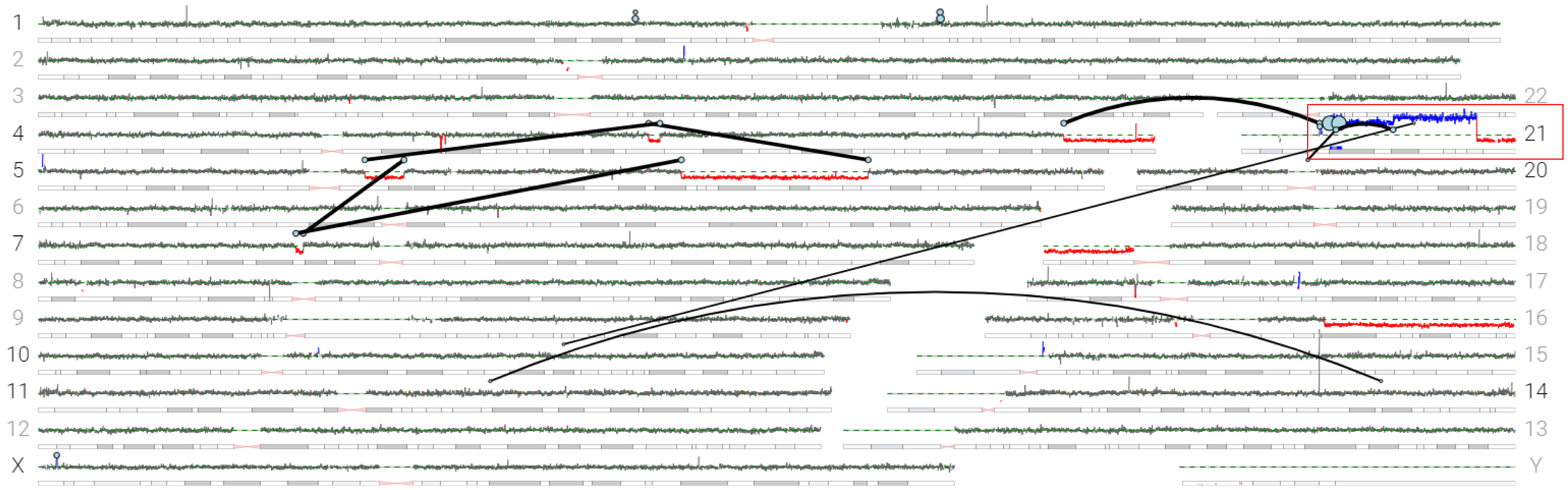

Supplementary Figure 6: Identification of additional abnormalities by MPseq. A. Junction plot (top) and focal U-shaped plot (bottom) of a *KMT2A* tandem duplication (chr11q23.3:118449083-118485572x3, GRCh38) from case NK-34. **B.** Whole genome plot showing iAMP21 (red box) of case 5q-65. Red lines indicate copy number losses and blue lines indicate copy number gains, black lines indicate junctions between chromosomes.
