# Supplementary material for: Prospective evaluation of genome sequencing to compare conventional cytogenetics in acute myeloid leukemia
Source: Blood Cancer J. 2023 Sep 6;13(1):138. doi: 10.1038/s41408-023-00908-5 (PMC10482828; doi:10.1038/s41408-023-00908-5)
Supplement: Supplementary file 3 — Supplementary Tables [file 41408_2023_908_MOESM3_ESM.docx]

**Supplementary Table 1: Demographic characteristics of cohort**

|  | **All**  **(105, 100%)** | **NK**  **(37, 35%)** | **5q and/or 7q deletion (25, 24%)** | **Simple**  **(7, 7%)** | **NUP98r**  **(6, 6%)** | **+8**  **(6, 6%)** | ***KMT2Ar***  **(5, 5%)** | **Atypical CK**  **(5, 5%)** | **Other**  **(14, 13%)** |
| --- | --- | --- | --- | --- | --- | --- | --- | --- | --- |
| **Median age (years)** | 65 | 68 | 67 | 71 | 39 | 72 | 32 | 55 | 56 |
| **Gender (% male)** | 58 (55%) | 20 (54%) | 13 (52%) | 4 (57%) | 5 (83%) | 4 (67%) | 3 (60%) | 1 (20%) | 8 (57%) |
| **De novo AML (%)** | 55 (52%) | 22 (59%) | 10 (40%) | 1 (14%) | 3 (50%) | 4 (67%) | 3 (60%) | 2 (40%) | 10 (71%) |
| **AML-MRC (%)** | 23 (22%) | 8 (22%) | 7 (28%) | 3 (43%) | 0 (0%) | 1 (17%) | 1 (20%) | 2 (40%) | 1 (7%) |
| **Relapsed AML (%)** | 21 (20%) | 6 (16%) | 5 (20%) | 2 (29%) | 3 (50%) | 1 (17%) | 1 (20%) | 1 (20%) | 2 (14%) |
| **Therapy related AML (%)** | 6 (6%) | 1 (3%) | 3 (12%) | 1 (14%) | 0 (0%) | 0 (0%) | 0 (0%) | 0 (0%) | 1 (7%) |

105 samples with patient self-reported gender, age at time of genomic testing, and morphologic diagnosis in relation to cytogenetic subtypes. Other category (n=14) includes the subgroups: t(9;11)(p22;q23) (n=2), inv(16)(p13.1q22) (n=3), inv(3)(q21q26) (n=3), t(15;17)(q24;q21) (n=4), t(6;9)(p23.3;q34.1) (n=1), KAT6A rearrangement (n=1).

**Supplementary Table 2: Karyotype results**

| **Case ID** | **Sex** | **Age** | **Disease Classification** | **Karyotype** |
| --- | --- | --- | --- | --- |
| NK-1 | F | 68 | De novo AML | 46,XX[20] |
| NK-2 | M | 52 | Relapsed AML | 46,XY[8] |
| NK-3 | M | 74 | De novo AML | 46,XY[20] |
| NK-4 | M | 78 | AML-MRC | 46,XY[20] |
| NK-5 | M | 55 | De novo AML | 46,XY[20] |
| NK-6 | F | 58 | De novo AML | 46,XX[20] |
| NK-7 | F | 77 | AML-MRC | 46,XX[20] |
| NK-8 | F | 9 | De novo AML | 46,XX[20] |
| NK-9 | F | 34 | De novo AML | 46,XX[20] |
| NK-10 | M | 78 | De novo AML | 46,XY[20] |
| NK-11 | M | 71 | Relapsed AML | 46,XY[20] |
| NK-12 | F | 84 | Relapsed AML | 46,XX[20] |
| NK-13 | F | 65 | AML-MRC | 46,XX[20] |
| NK-14 | F | 72 | AML-MRC | ND, FISH normal |
| NK-15 | F | 68 | AML-MRC | 46,XX[20] |
| NK-16 | M | 68 | De novo AML | 46,XY[20] |
| NK-17 | M | 73 | De novo AML | 46,XY[20] |
| NK-18 | F | 71 | AML-MRC | 46,XX[20] |
| NK-19 | F | 39 | De novo AML | 46,XX[20] |
| NK-20 | M | 51 | Relapsed AML | 46,XY[18] |
| NK-21 | M | 90 | Therapy-related AML | 46,XY[20] |
| NK-22 | M | 65 | De novo AML | 46,XY[20] |
| NK-23 | M | 80 | De novo AML | 46,XY[20] |
| NK-24 | M | 84 | AML-MRC | 46,XY[20] |
| NK-25 | M | 30 | De novo AML | 46,XY[20] |
| NK-26 | M | 79 | De novo AML | 46,XY[20] |
| NK-27 | F | 74 | De novo AML | 46,XX[15] |
| NK-28 | F | 71 | De novo AML | 46,XX[20] |
| NK-29 | M | 70 | De novo AML | 46,XY[20] |
| NK-30 | M | 65 | AML-MRC | 46,XY[20] |
| NK-31 | M | 64 | De novo AML | 46,XY[20] |
| NK-32 | F | 38 | Relapsed AML | 46,XX[20] |
| NK-33 | F | 66 | De novo AML | 46,XX[20] |
| NK-34 | F | 74 | Relapsed AML | 46,XX[20] |
| NK-35 | M | 38 | De novo AML | 45,X,-Y[11]/46,XY[9] |
| NK-36 | F | 53 | De novo AML | 46,XX[20] |
| NK-37 | M | 28 | De novo AML | 46,XY[20] |
| 7q-53 | F | 67 | AML-MRC | 44,XX,-7,-17,der(18)t(17;18)(q11.2;q11.2)[18]/46,XX[2] |
| 7q-54 | M | 74 | AML-MRC | 45,XY,-7[7]/45,idem,del(12)(p11.2p13)[13] |
| 7q-55 | F | 58 | Therapy-related AML | 46,XX,der(7)t(7;11)(q11.2;q13)[20] |
| 7q-56 | F | 38 | Relapsed AML | 46,XX,add(3)(q27),del(7)(q22),add(12)(p11.2)[cp18]/46,idem,t(1;2)(q21;p13)[2] |
| 7q-57 | F | 82 | AML-MRC | 45,XX,-7[18]/46,sl,+8[2] |
| 7q-58 | M | 66 | AML-MRC | 45,XY,-7[12]/47,XY,+8[1]/46,XY[7] |
| 7q-59 | F | 55 | Relapsed AML | 46,XX,del(7)(q21q36)[11]//46,XY[9] |
| 5q-65 | F | 72 | AML-MRC | 45,XX,add(4)(q21),add(5)(q11.2),add(7)(p13),der(16)t(16;18)(q13;q11.2),-18,add(21)(q22)[14]/46,XX,t(11;17)(q23;q11.2)[6] |
| 5q-66 | M | 56 | Relapsed AML | 44,X,-Y,i(5)(p10), 16[2]/44,idem,der(4)t(4;16)(q13;q22),der(6)t(4;6)(q13;q23)[8]//46,XX[10] |
| 5q-67 | M | 72 | De novo AML | 44-45,XY,+der(4)add(4)(p12)t(4;6)(q35;p21.3),der(4)add(4)(p12)t(4;6)(q35;p21.3),-5,-6,+add(8)(p23),-11,add(11)(p13),-17, add(18)(q21),del(20)(q11.2q13.3),+0-mar[cp20] |
| 5q-68 | M | 73 | AML-MRC | 56-65,XX,+X,+1,+der(2)add(2)(p11.2)add(2)(q35),add(3)(q21),+5,del(5)(q13q33)x2,+6,+6,+8,+8,+9,+der(11)t(11;12)(p13;q15),add(12)(q24.3),+13,+14,+15,+16,+18,+add(18)(q23),+20,add(21)(p11.2),+22,add(22)(p11.2),+1-3mar[cp20] |
| 5q-147 | F | 81 | De novo AML | 42-51,XX,add(3)(p21),-5,-6,add(7)(p11.2),+8,-15,-16,del(17)(p11.2),-18,add(21)(p11.2),der(21)t(15;21)(q22;q22),+22,+0-5mar[cp19]/46,XX[1] |
| 5q-148 | F | 49 | Relapsed AML | 44-46,XX,dic(X;7)(p11.2;p13),add(3)(q26.2),add(4)(q21),add(5)(q11.2),der(7;17)(q10;q10),del(9)(q21q32),+21[cp17]//46,XY[3] |
| 5q/7q-84 | F | 71 | Therapy related AML | 43-44,XX,?dic(3;14)(q29;p11.2),add(5)(q31),-7,der(9;17)(q10;q10),+mar[cp20] |
| 5q/7q-85 | M | 58 | AML-MRC | 44-45,XY,add(4)(q21),add(5)(q13),add(6)(q15),-7,-10,add(10)(p13),-17,add(20)(p13),add(21)(q22),+1-2mar[cp20] |
| 5q/7q-87 | M | 59 | De novo AML | 44,XY,der(5;17)(p10;q10),del(7)(q22),del(8)(p21),del(9)(q13q22),add(12)(p11.2),-15,-20,+mar[9]/45,idem,+del(8)(p21)[6]/46,XY[5] |
| 5q/7q-88 | M | 58 | De novo AML | 44,XY,der(1;16)(q10;p10),der(5)t(1;5)(p22;q21),add(7)(q11.2),inv(12)(q13q15),del(13)(q12q22),add(13)(q32),der(18)t(1;18)(p22;p11.2),del(20)(q11.2q13.1),-22[9]/44,XY,der(1;16)(q10;p10),der(5)t(1;5)(p22;q21),-6,-7,add(12)(p13),+16,-18,del(20)(q11.2q13.1),-22,+r,+mar[9]/44,XY,der(1;16)(q10;p10),der(5)t(1;5)(p22;q21),-7,del(13)(q12q22),-18,del(20)(q11.2q13.1),-22,+mar[2] |
| 5q/7q-89 | M | 80 | De novo AML | 45-46,inv(2)(p11.2q13)?c,del(5)(q13q33),del(6)(q25),-7,dic(12;?)(p11.2;?),add(17)(p11.2),add(21)(q22),+0-1mar[19]/ 46,XY,inv(2)(p11.2q13)?c[1] |
| 5q/7q-90 | M | 77 | Relapsed AML | 43-44,XY,der(5;17)(p10;q10),add(7)(q11.2),-13,-17,add(19)(p13.3),+mar[cp4]/41-44,sl,dic(15;21)(p11.2;p11.2)[cp12]/ 43,sl,i(11)(q10)[2]/46,XY[1] |
| 5q/7q-91 | M | 87 | De novo AML | 44,XY,del(5)(q31q35),-6,add(7)(q11.2),idic(11)(p11.2),add(17)(p11.2),psu dic(19;6)(p13.1;p23),-20[8]/ 43,XY,del(5)(q31q35),-6,add(7)(q11.2),der(11;18)(q10;q10),add(17)(p11.2),psu dic(19;6)(p13.1;p23),-20[7]/,46,XY[5] |
| 5q/7q-92 | F | 65 | De novo AML | 43-44,XX,add(1)(q21),-2,add(2)(q35),del(3)(p11),-4,add(5)(q11.2),-7,add(12)(p11.2),-14,+16,add(16)(p13.3),-17,add(17)(p11.2),-18,add(19)(q13.1),add(19)(q13.3),-20,add(21)(q22),+1-2r,+2-3mar[cp18]/46,XX[2] |
| 5q/7q-93 | M | 29 | De novo AML | 46-48,add(X)(p22.3),-Y,-5,add(10)(q26),-11,+13,+add(14)(q32),+1-2r,+mar[cp14]/46,XY[6] |
| 5q/7q-94 | F | 63 | Therapy related relapsed AML | 45,XX,der(5;7)(p10;p10),add(12)(p11.2),add(17)(p11.2)[5]/45,sl,del(6)(q11)[13]/46,XX[2] |
| 5q/7q-95 | M | 71 | De novo AML | 46,XY,add(3)(q21),der(12)t(3;12)(q26;p13)[3]/39-45,sl,add(1)(q21),-add(3),-5,-7,add(7)(q32),+8,add(11)(q23),-12,-16,-21,-20,+22,+0-2mar[cp17] |
| 5q/7q-96 | F | 74 | De novo AML | 46-51,XX,-3,dic(5;13)(q11.2;p11.2),del(7)(q22q34),der(10)t(3;10)(p11;q24),add(12)(p11.2),+2-7r,+0-2mar[cp20] |
| t(9;11)-104 | F | 55 | AML-MRC | 46,XX,t(9;11)(p22;q23)[11]/46,XX,idem,del(6)(p23)[7]/46,XX[2] |
| t(9;11)-105 | F | 60 | De novo AML | 46,XX,t(9;11)(p22;q23)[20] |
| inv(16)-106 | M | 56 | Relapsed AML | 46,XY,inv(16)(p13.1q22)[12]//46,XX[8] |
| inv(16)-107 | M | 24 | De novo AML | 46,XY,inv(16)(p13.1q22)[20] |
| inv(16)-108 | M | 75 | Relapsed AML | 59,XY,+Y,+3,+5,+8,+8,+12,+13,+14,+15,inv(16)(p13.1q22),+19,+20,+21,+22[20] |
| inv(3)-109 | F | 51 | AML-MRC | 46,XX,inv(3)(q21q26.2),t(8;9;22)(q22;q34;q11.2)[14]/46,sl,add(3)(q21)[6] |
| inv(3)-110 | M | 73 | De novo AML | 46,XY,inv(3)(q21q26.2)[20] |
| inv(3)-111 | M | 56 | De novo AML and multiple myeloma | 46,XY,inv(3)(q21q26.2)[7]/46,sl,inv(3)(q21q26.2)[13] |
| KMT2Ar-112 | F | 25 | De novo AML | 46,X,-X,add(3)(p21),add(5)(q11.2),-8,add(9)(q22),t(11;17)(q23;q21), add(12)(q13),add(13)(q12),-22,+3mar[3]//46,XY[17] |
| KMT2Ar-113 | F | 32 | De novo AML | 46,XX,t(11;19)(q23;p13.1)[19]/46,XX[1] |
| KMT2Ar-114 | M | 56 | De novo AML | 46,XY,t(6;11;10)(q27;q23;p13)[20] |
| KMT2Ar-115 | M | 73 | AML-MRC | 46,XY[20] |
| KMT2Ar-116 | M | 31 | Relapsed AML | 46-49,XY,del(1)(q11),add(3)(q21),-5,add(10)(p11.2),del(11)(q13q23),-14,add(19)(p13.1),+21,+2-5mar[cp12]/46,XY[8] |
| t(15;17)-117 | F | 65 | De novo AML | 46,X,add(X)(q22),t(15;17)(q24;q21)[20] |
| t(15;17)-118 | M | 31 | De novo AML | 46,XY,t(15;17)(q24;q21)[18]/46,XY[2] |
| t(15;17)-119 | F | 54 | De novo AML | 47,XX,+8,t(15;17)(q24;q21)[3]/48,sl,+21[16]/46,XX[1] |
| t(15;17)-120 | M | 69 | De novo AML | ND |
| t(6;9) DEK/NUP214-121 | M | 39 | De novo AML | 46,XY,t(6;9)(p23;q34)[9]/46,sl,del(7)(q32)[9]/46,sl,add(7)(q22)[2] |
| NUP98/KDM5A-122 | F | 1 | De novo AML | 48,XX,+6,t(11;17;12)(p11.2;q21;p13),+21[10]/48,sl,add(3)(p25)[8]/46,XX[2] |
| NUP98/KDM5A-123 | M | 69 | Relapsed AML | 46,XY[20] |
| NUP98/KDM5A-124 | M | 26 | De novo AML | 46,XY[20] |
| NUP98/KDM5A-125 | M | 2 | De novo AML | 46,XY,der(11)add(11)(p11.2)ins(11;?)(q13;?),der(12)t(11;12)(p15;q11)[7]/46,XY[13] |
| NUP98/NSD1-126 | M | 51 | Relapsed AML | 47,XY,+8[18]/46,XY[2] |
| NUP98/NSD1-127 | M | 68 | Relapsed AML | 48,XY,+8,+13[14]/46,XY[6] |
| KAT6Ar-128 | F | 50 | De novo AML | 46,XX[20] |
| Simple K-129 | F | 74 | AML-MRC | 46,XX,1-28dmin[14]/46,XX[6] |
| Simple K-130 | F | 78 | AML-MRC | 46,XX,del(11)(q13q23)[1]/47,XX,+4[1]/46,XX[18] |
| Simple K-131 | F | 71 | De novo AML | 46,XX[20] FISH identified 13q deletion |
| Simple K-132 | M | 33 | Relapsed AML | 46,XY,+7,t(10;17)(p13;q21)[19]//46,XX[1] |
| Simple K-133 | M | 72 | Therapy related AML | 46,XY,del(12)(p11.2p13),del(16)(q22)[11]/46,XY[9] |
| Simple K-134 | M | 71 | AML-MRC | 46,XY,del(3)(q13.2q25)[15]/46,XY[5] |
| Simple K-135 | M | 54 | Relapsed AML | 47,XY,+13[2]/46,XY[18] |
| Trisomy 8-136 | F | 21 | De novo AML | 47,XX,+8[15]/46,XX[5] |
| Trisomy 8-137 | F | 70 | De novo AML | 47,XX,+8[18]/46,XX[2] |
| Trisomy 8-138 | M | 46 | Relapsed AML | 47,XY,+8[1]/46,XY[19] |
| Trisomy 8-139 | M | 87 | De novo AML | 47,XY,+8[11]/48,idem,+13[9] |
| Trisomy 8-140 | M | 75 | AML-MRC | 47,XY,+8[3]/46,XY[17] |
| Trisomy 8-141 | M | 74 | De novo AML | 47,XY,+8[6]/46,XY[7] |
| Atypical CK-142 | M | 77 | De novo AML | 44-46,X,-Y,del(1)(p32p36.1),+4,-9,-10,+13,-16,+18,der(?)t(?;10)(?;q22),+0-3r,+0-1mar[cp18]/45,X,-Y[2] |
| Atypical CK-143 | F | 71 | AML-MRC | 46,XX,t(3;10)(q21;q24),add(5)(p13),t(9;17)(p22;p11.2),add(11)(q13)[20] |
| Atypical CK-144 | F | 55 | Relapsed AML | 47,X,del(X)(q24),t(1;10)(q32;q22),add(3)(p21),+add(11)(p11.2),t(15;17)(q11.2;q25)[7]//46,XY[13] |
| Atypical CK-145 | F | 50 | AML-MRC | 47,XX,der(13;21)(q10;q10),+add(21)(p11.2),+22[4]/48,sl,+add(21)[5]/48,sl,+der(13;21)[cp6]/48,sl,+der(13;21)x2,-21[3]/46,XX[2] |
| Atypical CK-146 | F | 25 | De novo AML | 47,XX,t(3;15)(p10;p10),t(6;9)(q13;q13),+8,t(8;17)(q24.1;q11.2)[20] |

Patient ID, age, gender, morphologic diagnosis and karyotype analysis.

**Table 3: ELN 2022 Risk assessment based on cytogenetics and molecular genetics data.**

| **Case ID** | **Karyotype** | **FISH** | **MPseq** | **Sequencing** | **Risk** |
| --- | --- | --- | --- | --- | --- |
| NK-1 | NK | Normal | NK | Mutated *NPM1* with *FLT3* ITD | I |
| NK-2 | NK | Normal | NK | WT *NPM1* with  *FLT3* ITD incomplete sequencing | U |
| NK-3 | NK | Normal | NK | Incomplete sequencing | U |
| NK-4 | NK | Normal | NK | Incomplete sequencing | U |
| NK-5 | NK | Normal | NK | WT *NPM1* without *FLT3-ITD;* mutated *SRSF2* | A |
| NK-6 | NK | Normal | NK | WT *NPM1* without *FLT3-ITD* | I |
| NK-7 | NK | Normal | NK | Mutated *CEBPA* | F |
| NK-8 | NK | Normal | NK | Mutated *CEBPA* | F |
| NK-9 | NK | Normal | NK | Mutated *CEBPA* | F |
| NK-10 | NK | Normal | NK | Mutated *NPM1* without *FLT3-ITD* | F |
| NK-11 | NK | Normal | NK | No pathogenic variants | I |
| NK-12 | NK | Normal | NK | No pathogenic variants | I |
| NK-13 | NK | Normal | NK | Mutated *NPM1* without *FLT3-ITD* | F |
| NK-14 | ND | Normal | NK | WT *NPM1* without *FLT3-ITD;* mutated *EZH2* | A |
| NK-15 | NK | Normal | NK | WT *NPM1* without *FLT3-ITD;* mutated *RUNX1, BCOR* | A |
| NK-16 | NK | Normal | NK | Mutated *CEBPA* | F |
| NK-17 | NK | Normal | NK | WT *NPM1* with *FLT3* ITD incomplete sequencing | U |
| NK-18 | NK | Normal | NK | WT *NPM1* without *FLT3-ITD;* mutated *RUNX1, BCOR* | A |
| NK-19 | NK | Normal | NK | Mutated *NPM1* without *FLT3* ITD | F |
| NK-20 | NK | Normal | NK | WT *NPM1* without *FLT3-ITD;* mutated *SRSF2* | A |
| NK-21 | NK | Normal | NK | WT *NPM1* with *FLT3* ITD*;* mutated U2AF1 | A |
| NK-22 | NK | Normal | NK | Mutated *CEBPA* | F |
| NK-23 | NK | Normal | NK | WT *NPM1* without *FLT3-ITD; m*utated *RUNX1, SRSF2*, *ASXL1, U2AF1* | A |
| NK-24 | NK | Normal | NK | WT *NPM1* without *FLT3-ITD;* mutated *ASXL1 and SRSF2* | A |
| NK-25 | NK | Normal | NK | WT *NPM1* with *FLT3* ITD incomplete sequencing | U |
| NK-26 | NK | Normal | NK | WT *NPM1* with *FLT3* ITD | I |
| NK-27 | NK | Normal | NK | Mutated *NPM1* with *FLT3* ITD | I |
| NK-28 | NK | Normal | NK | Mutated *NPM1* with *FLT3* ITD | I |
| NK-29 | NK | Normal | NK | WT *NPM1* with *FLT3* ITD*;* mutated *ASXL1* | A |
| NK-30 | NK | Normal | NK | WT *NPM1* with *FLT3* ITD*;* mutated *RUNX1, SRSF2, ASXL1* | A |
| NK-31 | NK | Normal | NK | WT *NPM1* without *FLT3* ITD*;* mutated *RUNX1, U2AF1, BCOR* and *TP53* | A |
| NK-32 | NK | Normal | NK | Mutated *CEBPA* | F |
| NK-33 | NK | Normal | NK | Incomplete sequencing | U |
| NK-34 | NK | Normal | NK | WT *NPM1* without *FLT3* ITD*;* mutated *ASXL1* | A |
| NK-35 | NK | Normal | NK | Mutated *CEBPA* | F |
| NK-36 | NK | Normal | NK | Mutated *NPM1* with *FLT3* ITD | I |
| NK-37 | NK | Normal | NK | No pathogenic variants | I |
| 7q-53 | 7q del/-7, TP53 del | 7q del/-7, TP53 del | 7q del/-7, TP53 del | Incomplete sequencing | A |
| 7q-54 | 7q del/-7 | 7q del/-7 | 7q del/-7 | WT *NPM1* without *FLT3-ITD;* mutated *RUNX1 and SRSF2* | A |
| 7q-55 | 7q del | 7q del | 7q del | WT *NPM1* without *FLT3-ITD;* mutated *EZH2* | A |
| 7q-56 | 7q del (CK) | 7q del | 7q del + ETV6::MECOM | WT *NPM1* without *FLT3-ITD;* mutated *RUNX1, SF3B1* | A |
| 7q-57 | 7q del/-7 | 7q del/-7 | 7q del/-7 | Incomplete sequencing | A |
| 7q-58 | 7q del/-7 | 7q del/-7 | 7q del/-7 | WT *NPM1* without *FLT3-ITD;* mutated *SRSF2* | A |
| 7q-59 | 7q del | 7q del | 7q del | No pathogenic variants | I |
| 5q-65 | 5q del with KMT2Ar | 5q del with KMT2Ar | 5q del  No KMT2Ar | Incomplete sequencing | A |
| 5q-66 | 5q del | 5q del | 5q del | WT *NPM1* without *FLT3-ITD;* mutated *TP53* and *ASXL1* | A |
| 5q-67 | 5q del, TP53 del | 5q del, TP53 del | 5q del, TP53 del | Incomplete sequencing | A |
| 5q-68 | 5q del | 5q del | 5q del | WT *NPM1* without *FLT3-ITD;* mutated *TP53* | A |
| 5q-147 | 5q del, TP53 del | 5q del | 5q del + atypical 7q del | No pathogenic variants | A |
| 5q-148 | 5q del, TP53 del | No recurrent AML subtype | 5q del + atypical 7q del | WT *NPM1* without *FLT3-ITD;* mutated *TP53* | A |
| 5q/7q-84 | 5q/7q del, TP53 del | 5q/7q del, TP53 del | 5q/7q del, TP53 del | WT *NPM1* without *FLT3-ITD;* mutated *TP53* | A |
| 5q/7q-85 | 5q/7q del, TP53 del | 5q/7q del | 5q/7q del | WT *NPM1* without *FLT3-ITD;* mutated *TP53, ASXL1* | A |
| 5q/7q-87 | 5q/7q del, TP53 del | 5q del, TP53 del | 5q/7q del, TP53 del | No pathogenic variants | A |
| 5q/7q-88 | 5q/7q del | 5q/7q del | 5q/7q del | WT *NPM1* without *FLT3-ITD;* mutated *TP53* | A |
| 5q/7q-89 | 5q/7q del, TP53 del | 5q/7q del | 5q/7q del | WT *NPM1* without *FLT3-ITD;* mutated *TP53* | A |
| 5q/7q-90 | 5q/7q del, TP53 del | 5q/7q del, TP53 del | 5q/7q del, TP53 del | WT *NPM1* without *FLT3-ITD;* mutated *TP53* | A |
| 5q/7q-91 | 5q/7q del, TP53 del | 5q/7q del, TP53 del | 5q/7q del, TP53 del | WT *NPM1* without *FLT3-ITD;* mutated *TP53* | A |
| 5q/7q-92 | 5q/7q del, TP53 del | 5q/7q del, TP53 del | 5q/7q del, TP53 del | WT *NPM1* without *FLT3-ITD;* mutated *TP53* | A |
| 5q/7q-93 | 5q/7q del | 5q/7q del | 5q/7q del | WT *NPM1* without *FLT3-ITD;* mutated *TP53* | A |
| 5q/7q-94 | 5q/7q del, TP53 del | 5q/7q del, TP53 del | 5q/7q del, TP53 del | WT *NPM1* without *FLT3-ITD;* mutated *TP53* and *RUNX1*, *U2AF1* | A |
| 5q/7q-95 | 5q/7q del | 5q/7q del, TP53 del | 5q/7q del, TP53 del | WT *NPM1* without *FLT3-ITD;* mutated *TP53* | A |
| 5q/7q-96 | 5q/7q del | 5q/7q del | 5q/7q del | WT *NPM1* without *FLT3-ITD;* mutated *TP53* | A |
| t(9;11)-104 | t(9;11), MLLT3::KMT2A | t(9;11), MLLT3::KMT2A | t(9;11), MLLT3::KMT2A | No pathogenic variants | I |
| t(9;11)-105 | t(9;11), MLLT3::KMT2A | t(9;11), MLLT3::KMT2A | t(9;11), MLLT3::KMT2A | Incomplete sequencing | I |
| inv(16)-106 | inv(16), MYH11::CBFB | inv(16), MYH11::CBFB | inv(16), MYH11::CBFB | No pathogenic variants | F |
| inv(16)-107 | inv(16), MYH11::CBFB | inv(16), MYH11::CBFB | inv(16), MYH11::CBFB | No pathogenic variants | F |
| inv(16)-108 | inv(16), MYH11::CBFB | inv(16), MYH11::CBFB | inv(16), MYH11::CBFB | No pathogenic variants | F |
| inv(3) with t(9;22)-109 | inv(3), GATA2/MECOM and t(9;22) | inv(3), GATA2/MECOM and t(9;22) | inv(3), GATA2/MECOM and t(9;22) | WT *NPM1* without *FLT3-ITD;* mutated *BCOR* | A |
| inv(3)-110 | inv(3), GATA2/MECOM | inv(3), GATA2/MECOM | inv(3), GATA2/MECOM | Incomplete sequencing | A |
| inv(3)-111 | inv(3), GATA2/MECOM | inv(3), GATA2/MECOM | inv(3), GATA2/MECOM | WT *NPM1* without *FLT3-ITD;* mutated *BCOR, SF3B1* | A |
| t(15;17)-117 | t(15;17), PML/RARA | t(15;17), PML/RARA | t(15;17), PML/RARA | Incomplete sequencing | N/A |
| t(15;17)-118 | t(15;17), PML/RARA | t(15;17), PML/RARA | t(15;17), PML/RARA | Incomplete sequencing | N/A |
| t(15;17)-119 | t(15;17), PML/RARA | t(15;17), PML/RARA | t(15;17), PML/RARA | Incomplete sequencing | N/A |
| t(15;17)-120 | ND | t(15;17), PML/RARA | t(15;17), PML/RARA | Incomplete sequencing | N/A |
| t(6;9) DEK:: NUP214-121 | t(6;9), DEK::NUP214 | t(6;9), DEK::NUP214 | t(6;9), DEK::NUP214 | Incomplete sequencing | A |
| KAT6Ar-128 | NK | Trisomy 8 or KAT6Ar | inv(8), KAT6A::SORBS3 | WT *NPM1* with *FLT3* ITD | I |
| KMT2Ar-112 | KMT2Ar | KMT2Ar, TP53 del | KMT2Ar, KMT2A::MLLT6, TP53 del | Incomplete sequencing | A |
| KMT2Ar-113 | KMT2Ar | KMT2Ar, KMT2A::ELL | KMT2Ar, KMT2A::ELL | Incomplete sequencing | A |
| KMT2Ar-114 | KMT2Ar | KMT2Ar, KMT2A::MLLT10 | KMT2Ar, KMT2A::MLLT10 | Incomplete sequencing | A |
| KMT2Ar-115 | NK | KMT2Ar, KMT2A::MLLT10 | KMT2Ar, KMT2A::MLLT10 | WT *NPM1* without *FLT3-ITD;* mutated *SRSF2* | A |
| KMT2Ar-116 | KMT2Ar | KMT2Ar, KMT2A::MLLT10 | KMT2Ar, KMT2A/MLLT10 | No pathogenic variants | A |
| NUP98r-122 | CK | NUP98r | CK with NUP98::KDM5A | Incomplete sequencing | A |
| NUP98r-123 | NK | NUP98r | NUP98::KDM5A | WT *NPM1* without *FLT3-ITD;* mutated *SRSF2* | A |
| NUP98r-124 | NK | NUP98r | NUP98::KDM5A | WT *NPM1* with *FLT3* ITD | I |
| NUP98r-125 | Simple K | NUP98r | NUP98::KDM5A | Incomplete sequencing | U |
| NUP98r-126 | Simple K | NUP98r | NUP98::NSD1 | WT *NPM1* with *FLT3* ITD | I |
| NUP98r-127 | Simple K | NUP98r | NUP98::NSD1 | Incomplete sequencing | U |
| Simple K-129 | Simple K | No recurrent AML subtype | Simple K | Incomplete sequencing | U |
| Simple K-130 | Simple K | Normal | Simple K | Mutated *CEBPA* | F |
| Simple K-131 | NK | No recurrent AML subtype | Simple K | Mutated *NPM1* without *FLT3-ITD* | F |
| Simple K-132 | Simple K | No recurrent AML subtype | Simple K | No pathogenic variants | I |
| Simple K-133 | Simple K | No recurrent AML subtype | Simple K | Mutated *CEBPA* | F |
| Simple K-134 | Simple K | No recurrent AML subtype | Simple K | WT *NPM1* with *FLT3-ITD;* mutated *ASXL1, SRSF2* | A |
| Simple K-135 | Simple K | No recurrent AML subtype | Simple K | WT *NPM1* with *FLT3-ITD;* mutated *BCOR* | A |
| Trisomy 8-136 | Simple K | Trisomy 8 | Simple K | No pathogenic variants | I |
| Trisomy 8-137 | Simple K | Trisomy 8 | Simple K | Incomplete sequencing | U |
| Trisomy 8-138 | Simple K | Trisomy 8 | Simple K | Incomplete sequencing | U |
| Trisomy 8-139 | Simple K | Trisomy 8 | Simple K | Incomplete sequencing | U |
| Trisomy 8-140 | Simple K | Trisomy 8 | Simple K | WT *NPM1* without *FLT3-ITD;* mutated *RUNX1, ASXL1, U2AF1* | A |
| Trisomy 8-141 | Simple K | Trisomy 8 | Simple K | WT *NPM1* without *FLT3-ITD;* mutated *ASXL1, U2AF1* | A |
| Atypical CK-142 | Atypical CK | No recurrent AML subtype | Atypical CK | WT *NPM1* without *FLT3-ITD;* mutated *ZRSR2* | A |
| Atypical CK-143 | Atypical CK | No recurrent AML subtype | Atypical CK | WT *NPM1* with *FLT3-ITD;* mutated *RUNX1, SRSF2* | A |
| Atypical CK-144 | Atypical CK | No recurrent AML subtype | Atypical CK | WT *NPM1* without *FLT3-ITD;* mutated *BCOR, EZH2* | A |
| Atypical CK-145 | Atypical CK | No recurrent AML subtype | Atypical CK | No pathogenic variants | A |
| Atypical CK-146 | Atypical CK | No recurrent AML subtype | Atypical CK | WT *NPM1* without *FLT3-ITD;* mutated *STAG2* | A |

ELN 2022 risk assessment (adverse, intermediate or favorable) based on cytogenetics and molecular genetics data. Incomplete sequencing indicated when sequencing was not performed for *NPM1, CEBPA, FLT3, RUNX1, TP53 or ASXL1, BCOR, EZH2, SF3B1, SRSF2, STAG2, U2AF1 and ZRSR2.* When pathogenic or likely pathogenic SNVs were not identified in these genes, no pathogenic/likely pathogenic SNV is indicated. The t(15;17) is not included in risk stratification. F: Favorable; I, intermediate; A: Adverse. When risk assessment could not be determined, a U is indicated. Cases that were discordant between MPseq and karyotype+FISH are highlighted.
