# Supplementary material for: Prospective evaluation of genome sequencing to compare conventional cytogenetics in acute myeloid leukemia
Source: Blood Cancer J. 2023 Sep 6;13(1):138. doi: 10.1038/s41408-023-00908-5 (PMC10482828; doi:10.1038/s41408-023-00908-5)
Supplement: Supplementary file 4 — Supplementary Table 4 [file 41408_2023_908_MOESM4_ESM.pdf]

| Supplementary Table 4: Cases with additional information identified by MPseq. AML genes associated with an SV, deletion or gain indicated in red that were not recognized by karyotype or FISH. This may include identification of fusion partner genes. | | | | |
|---|---|---|---|---|
| **Paper ID** | **MPseq identified new info** | **AML Genes Rearranged by SVs** | **AML Genes in Deletions** | **AML Genes in Gains** |
| NK-1 | No | N/A | N/A | N/A |
| NK-2 | Yes | None | **FAT1** | **MYB, CEP43 BCR** |
| NK-3 | No | N/A | N/A | N/A |
| NK-4 | No | N/A | N/A | N/A |
| NK-5 | No | N/A | N/A | N/A |
| NK-6 | No | N/A | N/A | N/A |
| NK-7 | Yes | N/A | **DNMT3A, CEBPA** | N/A |
| NK-8 | No | N/A | N/A | N/A |
| NK-9 | No | N/A | N/A | N/A |
| NK-10 | No | N/A | N/A | N/A |
| NK-11 | Yes | N/A | N/A | **CCDC26** |
| NK-12 | No | N/A | N/A | N/A |
| NK-13 | No | N/A | N/A | N/A |
| NK-14 | N/A | N/A | N/A | N/A |
| NK-15 | No | N/A | N/A | N/A |
| NK-16 | No | N/A | N/A | N/A |
| NK-17 | No | N/A | N/A | N/A |
| NK-18 | No | N/A | N/A | N/A |
| NK-19 | Yes | N/A | **NF1, SUZ12** | **CCDC26** |
| NK-20 | No | N/A | N/A | N/A |
| NK-21 | No | N/A | N/A | N/A |
| NK-22 | No | N/A | N/A | N/A |
| NK-23 | No | N/A | N/A | N/A |
| NK-24 | No | N/A | N/A | N/A |
| NK-25 | No | N/A | N/A | N/A |
| NK-26 | No | N/A | N/A | N/A |
| NK-27 | No | None | N/A | N/A |
| NK-28 | Yes | N/A | **NF1** | N/A |
| NK-29 | No | N/A | N/A | N/A |
| NK-30 | No | N/A | N/A | N/A |
| NK-31 | No | N/A | N/A | N/A |
| NK-32 | No | N/A | N/A | N/A |
| NK-33 | No | N/A | N/A | N/A |
| NK-34 | Yes | N/A | N/A | **KMT2A partial tandem dup** |
| NK-35 | No | N/A | N/A | N/A |
| NK-36 | No | N/A | N/A | N/A |
| NK-37 | No | N/A | N/A | N/A |

| | | | | |
|---|---|---|---|---|
| 7q-53 | Yes | None | **FHIT,** IKZF1, CUX1, KMT2E, KMT2C, EZH2, TP53, MYH1, **SETBP1** | N/A |
| 7q-54 | No | N/A | IKZF1, CUX1, KMT2E, KMT2C, EZH2 PTEN ETV6, CDKN1B AEBP2 | N/A |
| 7q-55 | Yes | **CCDC26::BCL11B** | CUX1, KMT2E, KMT2C, EZH2 | **MYB,** KMT2A, CBL**, CCDC26** |
| 7q-56 | Yes | **MECOM::ETV6** | EZH2, CUX1, KMT2E, **NF1** | N/A |
| 7q-57 | No | None | IKZF1, CUX1, KMT2E, KMT2C, EZH2 | MYC, TRPS1, RAD21, RUNX1T1, KAT6A,FGFR1, [illegible] |
| 7q-58 | No | None | IKZF1, CUX1, KMT2E, KMT2C, EZH2 | N/A |
| 7q-59 | Yes | None | CUX1, KMT2E, KMT2C, EZH2 | KMT2A, CBL |
| 5q-65 | Yes | None | **TET2, FAT1**, APC, EGR1, CBFB, ZC3H1B | **iAMP21** |
| 5q-66 | Yes | None | FIP1L1, CHIC2,PDGFRA, KIT, APC, EGR1, DIAPH1, NR3C1, CSNK1A1, CSF1R, PDGFRB, RPS14, SPARC, NPM1, DDX41, CREBBP, MYH11, CBFB, ZC3H18, **NF1,** SUZ12 | TERT |

| | | | | |
|---|---|---|---|---|
| 5q-67 | Yes | None | APC, EGR1, DIAPH1, NR3C1, CSNK1A1, CSF1R, PDGFRB, RPS14, SPARC, NPM1, NUP98, TP53, **NF1,** SUZ12, RARA | FIP1L1, CHIC2, PDGFRA, KIT, TET2, FAT4, JARID2, DEK, MYB |
| 5q-68 | No | None | APC, EGR1, DIAPH1, NR3C1, CSNK1A1, CSF1R, PDGFRB, RPS14, SPARC | PRDM16, CSMD2, CSF3R, MPL, JAK1, RBM15, NRAS, ARNT, ENAH, TPO, DNMT3A, ASXL2, HOXD13, SF3B1, IDH1, INPP5D, TERT, NPM1, DDX41, JARID2, DEK, MYB, CEP43, PCM1, FGFR1, KAT6A, RUNX1T1, TRPS1, RAD21, MYC, JAK2, MLLT3, CDKN2A, CNTRL, ABL1, NUP214<br>NUP98, WT1, KMT2A, CBL<br>ZMYM2, FLT3, RB1<br>FOXN3, RCOR1<br>MGA, PML, IDH2<br>SETBP1<br>CALR, JAK3, CEBPA, U2AF2<br>ASXL1, L3MBTL1, SGK2<br>BCR, MRTFA, EP300 |
| 5q-148 | Yes | **MECOM::SATB1** | **ASXL2,** APC, CSNK1A1, CSF1R, PDGFRB, RPS14, SPARC, **IKZF1** | RUNX1, U2AF1 |
| 5q-147 | Yes | **RUNX1r** | **TET2,** FHIT, FOXP1, KMT2C, ETV6, CDKN1B, AEBP2 | **iAMP21** |

| | | | | |
|---|---|---|---|---|
| 5q/7q-84 | Yes | **MECOMr** | GATA2, RPN1, MLF1, MECOM, EGR1, DIAPH1, NR3C1, CSNK1A1, CSF1R, PDGFRB, RPS14, SPARC, NPM1, DDX41, CUX1, KMT2E, KMT2C, EZH2, TP53, **NF1** | MECOM<br><br>BCR, MRTFA, EP300 |
| 5q/7q-85 | Yes | **RUNX1r, ANKRD26r** | **TET2, FAT4, FAT1**, APC, EGR1, DIAPH1, NR3C1, CSNK1A1, CSF1R, PDGFRB, RPS14, SPARC, NPM1, DDX41, IKZF1, CUX1, KMT2E, KMT2C, EZH2, PRKG1, ETV6, CDKN1B, AEBP2, KRAS, **NF1**,SUZ12, RARA | N/A |
| 5q/7q-87 | Yes | **ASXL1r** | **TET2**, APC, EGR1, DIAPH1, NR3C1, CSNK1A1, CSF1R, PDGFRB, RPS14, SPARC, CUX1, KMT2E, EZH2, KMT2C, PCM1, MGA, CBFB, ZC3H18, TP53, **ASXL1**, L3MBTL1, SGK2 | **PRDM16, CSMD2, CSF3R<br>NPM1, DDX41<br>FGFR1, KAT6A, RUNX1T1, TRPS1, RAD21, MYC<br>CREBBP** |

| | | | | |
|---|---|---|---|---|
| 5q/7q-88 | Yes | None | APC, EGR1, DIAPH1, NR3C1, CSNK1A1, CSF1R, PDGFRB, RPS14, SPARC, JARID2, CEP43, CUX1, KMT2E, EZH2, KMT2C, ABL1, NUP214, ETV6, CDKN1B, RB1, RBBP6, CBFB, ZC3H18, **SETBP1**, SALL3, L3MBTL1, SGK2, BCR | **PRDM16, CSMD2, CSF3R,** MPL, JAK1, IKZF1 |
| 5q/7q-89 | Yes | None | APC, EGR1, DIAPH1, NR3C1, CSNK1A1, CSF1R, PDGFRB, RPS14, SPARC, IKZF1, CUX1, KMT2E, EZH2, KMT2C, AEBP2 | **PRDM16, CSMD2, CSF3R,** MPL |
| 5q/7q-90 | Yes | None | APC, EGR1, DIAPH1, NR3C1, CSNK1A1, CSF1R, PDGFRB, RPS14, SPARC, NPM1, DDX41, CUX1, KMT2E, EZH2, KMT2C, NUP98, **WT1**, ZMYM2, FLT3, RB1, TP53, MYO18A, NF1, SUZ12, RARA, PPM1D, SRSF2, CEBPA | KMT2A, CBL |

| | | | | |
|---|---|---|---|---|
| 5q/7q-91 | Yes | None | EGR1, DIAPH1, NR3C1, CSNK1A1, CSF1R, PDGFRB, RPS14, SPARC, JARID2, DEK, CUX1, KMT2E, EZH2, KMT2C, NUP98, **WT1,** TP53, MYO18A, NF1, SUZ12, CEBPA, L3MBTL1, SGK2 | KMT2A, CBL<br>CALR<br>JAK3<br>U2AF2 |
| 5q/7q-92 | Yes | **NF1::RAB11FIP3** | **FHIT, FOXP1**, FIP1L1, CHIC2, PDGFRA, KIT, APC, EGR1, DIAPH1, NR3C1, CSNK1A1, CSF1R, PDGFRB, RPS14, SPARC, IKZF1, CUX1, KMT2E, EZH2, KMT2C, AEBP2, KRAS, TP53, SALL3 | PRDM16, TET2, FAT4, FAT1 |
| 5q/7q-93 | Yes | **CBL** | APC, EGR1, DIAPH1, NR3C1, CSNK1A1, CSF1R, PDGFRB, RPS14, SPARC, CUX1, KMT2E, EZH2, KMT2C, **WT1** | SSBP2<br>MYB, CEP43<br>NUP98<br><br>KMT2A, CBL<br>ZMYM2, FLT3, RB1 |

| | | | | |
|---|---|---|---|---|
| 5q/7q-94 | Yes | **FHIT, MECOM::BCHE** | FHIT, APC, EGR1, DIAPH1, NR3C1, CSNK1A1, CSF1R, PDGFRB, RPS14, SPARC, NPM1, MYB, CEP43, IKZF1, CUX1, KMT2E, EZH2, KMT2C, ETV6, CDKN1B, AEBP2, TP53 | **MECOM amp, KRAS amp** |
| 5q/7q-95 | Yes | None | FHIT, FOXP1, GATA2, RPN1, TERT, SSBP2, APC, EGR1, DIAPH1, NR3C1, CSNK1A1, CSF1R, PDGFRB, RPS14, SPARC, NPM1, DDX41, CEP43, IKZF1, CUX1, KMT2E, EZH2, KMT2C, ETV6, CDKN1B, AEBP2, SOCS2, CREBBP, MYH11, RBBP6, CBFB, ZC3H18, TP53, **SETBP1**, SALL3, L3MBTL1, SGK2, RUNX1, U2AF1 | PCM1, FGFR1, KAT6A, RUNX1T1, TRPS1, RAD21, MYC KMT2A, CBL |
| 5q/7q-96 | Yes | **RPN1::MECOM + MECOMr** | GATA2, RPN1, MLF1 , SSBP2, APC, EGR1, DIAPH1, NR3C1, CSNK1A1, CSF1R, PDGFRB, RPS14, SPARC, NPM1, DDX41, CUX1, KMT2E, EZH2, KMT2C, ETV6, CDKN1B, AEBP2 | **MECOM amp, KRAS amp** |

| | | | | |
|---|---|---|---|---|
| t(9;11)-104 | No | MLLT3::KMT2A | None | None |
| t(9;11)-105 | No | MLLT3::KMT2A | N/A | N/A |
| inv(16)-106 | No | MYH11::CBFB | N/A | N/A |
| inv(16)-107 | No | MYH11::CBFB | N/A | N/A |
| inv(16)-108 | No | MYH11::CBFB | N/A | FHIT, FOXP1, GATA2, RPN1, MLF1, MECOM<br>TERT, APC, EGR1, DIAPH1, NR3C1, CSNK1A1, CSF1R, PDGFRB, RPS14, SPARC, NPM1, DDX41<br>PCM1, FGFR1, KAT6A, RUNX1T1, TRPS1, RAD21, MYC<br>ETV6, CDKN1B, AEBP2, KRAS, HMGA2, SOCS2, SH2B3, PTPN11<br>ZMYM2, FLT3, RB1<br>FOXN3, RCOR1<br>MGA, PML, IDH2<br>CALR, JAK3, CEBPA, U2AF2<br>ASXL1, L3MBTL1, SGK2<br>RUNX1, U2AF1<br>BCR, MRTFA, EP300 |
| inv(3)-109 | No | GATA2::MECOM, BCR::ABL1 | N/A | N/A |
| inv(3)-110 | Yes | GATA2::MECOM | **WT1** | N/A |

| | | | | |
|---|---|---|---|---|
| inv(3)-111 | No | GATA2::MECOM | N/A | FHIT, FOXP1, GATA2, RPN1, MLF1, MECOM<br>DHX15, FIP1L1, CHIC2, PDGFRA, KIT, TET2, FAT4, FAT1<br>TERT, APC, EGR1, DIAPH1, NR3C1, CSNK1A1, CSF1R, PDGFRB, RPS14, SPARC, NPM1, DDX41<br>IKZF1, CUX1, KMT2E, EZH2, KMT2C<br>MGA, PML, IDH2<br>TP53, MYO18A, NF1, SUZ12, RARA, PPM1D, SRSF2<br>CALR, JAK3, CEBPA, U2AF2<br>RUNX1, U2AF1 |
| KMT2Ar-112 | Yes | KMT2A::MLLT6, **MECOM::ZBBX** | KMT2A, **CBL**, TP53 | None |
| KMT2Ar-113 | No | KMT2A::ELL | N/A | N/A |
| KMT2Ar-114 | No | KMT2A::MLLT10 | N/A | N/A |
| KMT2Ar-115 | Yes | KMT2A::MLLT10 | **MRTFA, EP300** | N/A |
| KMT2Ar-116 | No | KMT2A::MLLT10 | None | TRPS1, RAD21, MYC<br>PPM1D, SRSF2<br>RUNX1, U2AF1 |
| t(15;17)-117 | No | PML::RARA | N/A | RUNX1T1, TRPS1, RAD21, MYC |
| t(15;17)-118 | No | PML::RARA | N/A | N/A |
| t(15;17)-119 | No | PML::RARA | N/A | PCM1, FGFR1, KAT6A, RUNX1T1, TRPS1, RAD21, MYC<br>RUNX1, U2AF1 |
| t(15;17)-120 | N/A | PML::RARA | N/A | N/A |
| t(6;9)-121 | No | DEK::NUP214 | N/A | N/A |
| NUP98/KDM5A-122 | Yes | **NUP98::KMD5A** | NUP98 (partial) | JARID2, DEK, MYB, CEP43<br>RUNX1, U2AF1 |
| NUP98/KDM5A-123 | Yes | **NUP98::KMD5A** | **RUNX1** | N/A |

| | | | | |
|---|---|---|---|---|
| NUP98/KDM5A-124 | Yes | **NSD1::NUP98** | N/A | N/A |
| NUP98/KDM5A-125 | Yes | **NUP98::KMD5A** | N/A | N/A |
| NUP98/NSD1-126 | Yes | **NSD1::NUP98, MLLT10::USH2A** | NUP98 (partial) | PCM1, FGFR1, KAT6A, RUNX1T1, TRPS1, RAD21, MYC |
| NUP98/NSD1-127 | Yes | **NSD1::NUP98** | N/A | PCM1, FGFR1, KAT6A, RUNX1T1, TRPS1, RAD21, MYC ZMYM2, FLT3, RB1 |
| KAT6A r-128 | Yes | **KAT6A::SORBS3** | N/A | N/A |
| Simple K-129 | Yes | N/A | N/A | **MYC amplification** |
| Simple K-130 | Yes | N/A | **DNMT3A, CEBPA** | DHX15, FIP1L1, CHIC2, PDGFRA, KIT, TET2, FAT4, FAT1 |
| Simple K-131 | No | N/A | RB1 | N/A |
| Simple K-132 | Yes | **ZMYND11::MBTD1** | N/A | IKZF1, CUX1, KMT2E, EZH2, KMT2C |
| Simple K-133 | Yes | N/A | **IKZF1** , ETV6, CDKN1B, RB1, CBFB, **CEBPA** | N/A |
| Simple K-134 | No | N/A | GATA2, RPN1 | N/A |
| Simple K-135 | No | N/A | N/A | N/A |
| Trisomy 8-136 | No | N/A | N/A | PCM1, FGFR1, KAT6A, RUNX1T1, TRPS1, RAD21, MYC |
| Trisomy 8-137 | No | N/A | N/A | PCM1, FGFR1, KAT6A, RUNX1T1, TRPS1, RAD21, MYC |
| Trisomy 8-138 | No | N/A | N/A | N/A |
| Trisomy 8-139 | No | N/A | N/A | PCM1, FGFR1, KAT6A, RUNX1T1, TRPS1, RAD21, MYC ZMYM2, FLT3, RB1 |
| Trisomy 8-140 | No | N/A | N/A | N/A |

| | | | | |
|---|---|---|---|---|
| Trisomy 8-141 | No | N/A | N/A | PCM1, FGFR1, KAT6A, RUNX1T1, TRPS1, RAD21, MYC |
| Atypical CK-142 | No | None | PRDM16, CSMD2, CSF3R, JAK2, MLLT3, CDKN2A, CNTRL, ABL1, NUP214, CBFB, ZC3H18 | SETBP1 |
| Atypical CK-143 | No | ATM::TARS | N/A | N/A |
| Atypical CK-144 | No | None | N/A | NUP98<br>KMT2A, CBL |
| Atypical CK-145 | Yes | N/A | **RUNX1** | ZMYM2, FLT3, RB1, U2AF1, BCR, MRTFA, EP300 |
| Atypical CK-146 | Yes | **NF1::TG** | None | PCM1, FGFR1, KAT6A, RUNX1T1, TRPS1, RAD21, MYC |
| | | | | |
